# Time-resolved X-ray spectroscopy of phenanthridine: elucidating the photodynamics of a nitrogen-containing polycyclic aromatic hydrocarbon

**DOI:** 10.1039/d5sc03745j

**Published:** 2025-10-20

**Authors:** Dorothee Schaffner, Kira Diemer, Xincheng Miao, Emil Karaev, Marco Flock, Katharina Theil, Constant Schouder, Audrey Scognamiglio, Lou Barreau, Lionel Poisson, Dennis Mayer, Andre Al Haddad, Antoine Sarracini, Gregor Knopp, Xinhua Xie, Patrick Hemberger, Kirsten Schnorr, Roland Mitric, Ingo Fischer

**Affiliations:** a Institute of Physical and Theoretical Chemistry, University of Würzburg Am Hubland 97074 Würzburg Germany roland.mitric@uni-wuerzburg.de ingo.fischer@uni-wuerzburg.de; b Université Paris-Saclay, CNRS, Institut des Sciences Moléculaires d'Orsay 91405 Orsay France; c Deutsches Elektronen-Synchrotron DESY Notkestrasse 85 22607 Hamburg Germany; d Laboratory for Femtochemistry and Synchrotron Radiation, Paul Scherrer Institute (PSI) 5232 Villigen Switzerland

## Abstract

The photophysics and photochemistry of isolated phenanthridine have been investigated by time-resolved UV pump/X-ray probe spectroscopy at the SwissFEL free-electron laser combined with computations. Phenanthridine serves as the example for a polycyclic aromatic nitrogen-containing hydrocarbon (PANH), a class of molecules of considerable interest in material science and astrochemistry. It was excited at 268 nm into the bright 2ππ* state. The dynamics was subsequently probed by time-resolved X-ray photoelectron (TR-XPS) and X-ray absorption (TR-XAS) spectroscopy at the nitrogen 1s edge. Two time constants of *τ*_1_ ≈ 0.3 ps and *τ*_2_ ≈ 3 ps were determined. The excited-state dynamics was simulated using the trajectory surface hopping method and computed TR-XAS to support the band assignments. The study reveals a sequential decay to the electronic ground state *via* internal conversion. Spectra recorded over longer delay times indicate a dissociation on a time scale of several hundred picoseconds.

## Abstract 2

Phenanthridine serves as a model for the photochemistry of nitrogen-containing polycyclic aromatic hydrocarbons (PANH). Here, it has been investigated by time-resolved X-ray spectroscopy using free electron laser radiation.

## Graphical abstract

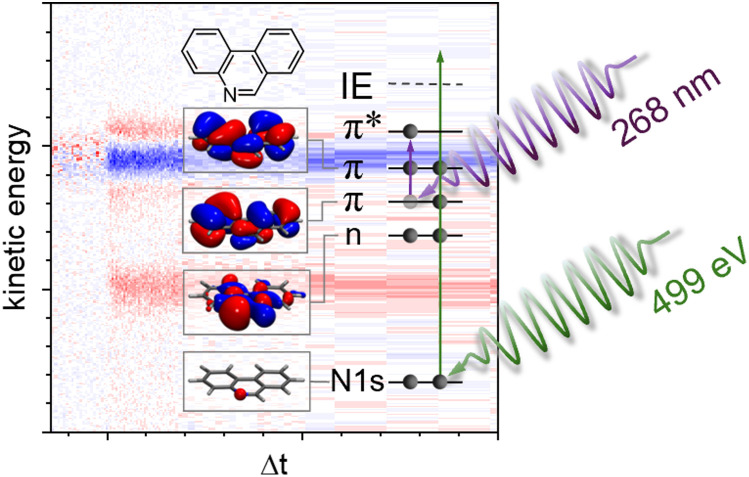

## 1 Introduction

**Fig. 1:**
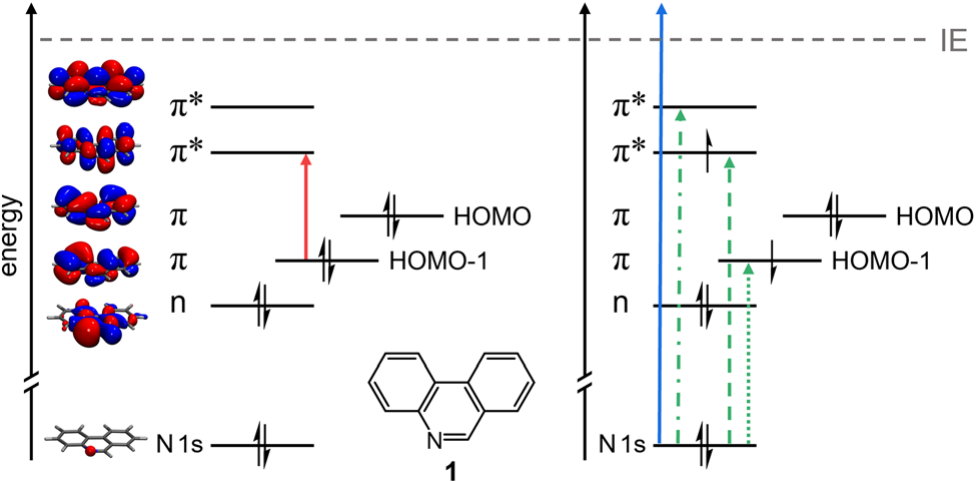
Scheme of the electronic processes in phenanthridine 1 investigated in this work. In the pump step (left), the molecule is valence excited into the S_3_ state. In the probe step (right), a N 1s electron is subsequently either ionized (TR-XPS, solid blue line) or excited (TR-XAS, dashed and dotted green lines) into a half filled or empty molecular orbital (MO). Note that the orbital energies are not drawn to scale. The depicted MOs are obtained at the ωB97X-D/def2-SV(P) level of theory.

In this manuscript, we present a time-resolved X-ray photoelectron and photoabsorption spectroscopy study of the nitrogen-doped polycyclic aromatic hydrocarbon (PANH) phenanthridine 1, see Fig. 1. PANHs currently attract considerable attention due to their role in several scientific areas. In astrochemistry, PANHs are considered as potential carriers of the Unidentified Infrared Bands (UIBs) that are observed in the interstellar medium.^1^ In particular, the characteristic emission band around 6.2 μm cannot be assigned to pure hydrocarbons,^2^ thus PANHs are currently investigated as alternative carriers. Furthermore, they are also assumed to be formed in the atmospheric chemistry of Titan, a moon of Saturn.^3^ In materials science and optoelectronics, PANHs are investigated as potential electron conductors,^4^ because the substitution of carbon by nitrogen allows the electronic properties of acenes to be modified.^5^ Thus, they potentially complement their pure hydrocarbon counterparts (PAHs), which are employed as hole conductors. Substitution of carbon by heteroatoms also permits modulating excited electronic state energies and thus finding new candidates for singlet fission materials, a process that has the potential to improve the efficiency of solar cells.^6,7^ Recent theoretical studies on the dinitrogen-doped *N*,*N*′-dihydroanthracenes and phenanthrenes have identified a number of PANHs as promising candidates for this process.^8^ First studies on aza-substituted TIPS-pentacene confirmed the assumption.^9^

The rich photophysics and photochemistry of PANHs challenge both the experimental and computational investigation of the excited-state dynamics. Only limited experimental data are available for PANHs, mainly originating from studies in solution or cold matrices.^10–12^ Accurate gas phase data that provide insight into the intrinsic properties of these molecules are even more scarce. Compared to their pure hydrocarbon counterparts, their photochemistry is significantly complicated by the presence of additional low-lying nπ* states due to possible excitation of the nitrogen lone pair electrons. These states open additional pathways for nonradiative deactivation. For example, nπ* states can potentially enhance internal conversion rates and could lead to more efficient intersystem crossing (ISC) according to the El Sayed rules.^13^

To distinguish electronic states in the cascade of nonradiative relaxation processes, time-resolved photoionization and photoelectron spectroscopy (TR-PES) combined with high-level theory have become the gold standard for investigating molecular dynamics in the gas phase.^14–17^ However, employing visible or UV probe pulses comes with a drawback: the relaxation process often includes transient structures with electronic configurations that correspond to inner valence excitations, which are preferentially ionized to excited electronic states of the cation. Since the ionization energy increases strongly for accessing excited ionic states, intermediate states can no longer be ionized and the subsequent steps in the deactivation cannot be identified experimentally. In particular, singlet or triplet nπ* states that correspond to excited ionic states in photoionization, as well as the vibrationally highly excited electronic ground state, cannot be efficiently ionized with UV pulses. Usually, the probe is chosen to limit resonant ionization from the neutral ground state, so Franck–Condon (FC) factors for photoionization to the cationic ground state are small. Thus, a UV probe usually does not provide full information on the transiently populated excited states. In contrast, the use of extreme ultraviolet (XUV) or soft X-ray probe pulses circumvents these issues, with the additional advantage that inner-shell electrons are highly sensitive to local structural and electronic changes.^18^ Recent years have seen a growth in soft X-ray probe techniques, concomitant with the increasing availability of free-electron lasers (FELs) as well as table-top X-ray sources based on high-order harmonic generation. Time-resolved X-ray absorption spectroscopy (TR-XAS) is a rather general method and has been applied in a number of studies in the gas phase and in solution.^19–22^ Time-resolved X-ray photoelectron spectroscopy (TR-XPS) on the other hand acquired popularity, especially when a heteroatom is present in the molecule.^23–27^ With this method, a change in the electron binding energy *E*_B_ upon ionization from electronically excited states (excited state chemical shift, ESCS) has been reported, and serves to gain insight into the dynamics.^28^ This shift is caused by the dependence of *E*_B_ on the local charge density and its variation in the excited electronic state. In thiouracil,^28,29^ ionization of the sulfur 2p electrons was shown to be sensitive to the change in electronic character at the sulfur atom as a function of time. The experiments outlined the role of the ^1^(nπ*) state, which acts as a doorway state for ISC to the triplet manifold. As a further probe method, time-resolved Auger–Meitner spectroscopy is particularly useful when investigating dissociation processes and was employed in studies of the dynamics of thymine and CS_2_.^20,30,31^

Phenanthridine 1 is an excellent model to investigate PANH dynamics, because some prior information is available. We recently reported multiphoton ionization spectra of three azaphenanthrenes, including 1 as well as benzo[*f*]quinoline and benzo[*h*]quinoline, combined with simulations based on *ab initio* computations.^32^ It was found that two ππ* and one nπ* states lie within 0.3 eV or less. In the present work, we employ TR-XPS and TR-XAS at the N 1s edge to monitor the deactivation processes in 1 after excitation at 268 nm and support the experiments by trajectory-based nonadiabatic dynamics simulations along with computed TR-XAS. This permits us to probe the changes occurring at the nitrogen site and to assess in particular the role of low-lying nπ* states, which have a small ionization probability to the ionic ground state in valence photoionization probe experiments.

## 2 Results

In the X-ray probe experiments discussed below, 1 is excited at 268 nm (4.63 eV). In agreement with our previous work,^32^ time-dependent density functional theory (TDDFT) calculations revealed that the lowest three excited singlet states are energetically accessible, see Table 1, (a). To characterize the adiabatic states S_1_ to S_3_, the natural transition orbitals (NTOs) for each state and the oscillator strengths were determined. As depicted in Fig. S1 in the SI, the S_1_ and S_3_ states have mixed ππ* character, whereas S_2_ is a pure nπ* transition. Consistent with this characterization, the computed oscillator strengths *f* indicate a dark S_2_ state (nπ*), and bright S_1_ and S_3_ states. These findings were supported by a high-level equation-of-motion coupled-cluster calculation with singles and doubles (EOM-CCSD), see Table 1, (b). As is evident from the ensemble spectrum shown in Fig. S3, the S_2_ state acquires intensity due to vibronic interaction outside the Franck–Condon region. Previous REMPI (resonance-enhanced multiphoton ionization, see Fig. S4) work showed a vibrationally resolved electronic spectrum with an origin at 3.67 eV that was assigned to the S_1_ ← S_0_ transition.^32,33^ At about 4.2 eV, a second transition sets in which is broad and unstructured, assigned to the second bright ππ* state.^32^ However, intensity perturbations in the spectrum have been identified by comparison with simulations, which indicate mixing of electronic states.

**Table 1:** Vertical excitation energies *E*_exc_ and oscillator strengths *f* for the lowest excited singlet states and the corresponding dominant transition characters

State	*E*_exc_/eV	*f*
**(a) ωB97X-D/def2-SV(P)**		
S_1_ (ππ*)	4.40	0.0146
S_2_ (nπ*)	4.53	0.0017
S_3_ (ππ*)	4.73	0.0456
S_4_ (ππ*)	5.37	0.1112

**(b) EOM-CCSD/cc-pVDZ**		
S_1_ (ππ*)	4.29	0.0072
S_2_ (nπ*)	4.80	0.0020
S_3_ (ππ*)	5.11	0.0233
S_4_ (ππ*)	5.28	0.0143

Initially, ps- and fs-time-resolved photoelectron spectroscopy (TR-PES) experiments using valence photoionization as a probe were carried out, as described in Fig. S5 and S6 in the SI. In the ps-experiments, previously identified S_1_ vibronic bands were excited with tunable radiation and ionized using either 351 nm or 263 nm probe light. While a ns-lifetime was observed at the origin at 3.670 eV (337.8 nm), a rapid decay on the ps-scale is visible for excited vibronic states. Time constants ranging from 100 ps at +0.235 eV (317.5 nm) down to 10 ps at +0.677 eV (285.2 nm) have been found.^34^ A signal offset at long delay times was visible, indicating that the final state of the deactivation can be ionized. In fs-TR-PES using 800 nm multiphoton ionization probe, a monoexponential decay with *τ* ≈ 7 ps was confirmed at 280 nm (not shown). However, at 266 nm excitation, the laser studies revealed a very different behavior. The time-resolved photoelectron signal could be adjusted by a biexponential decay. A sequential model was employed for the fit. We applied a two-step deactivation *A* → *B* → *C*, following the procedure outlined in Section S10 in the SI,^35^ and obtained two time constants, *τ*_1_ = 270 ± 50 fs and *τ*_2_ = 4.35 ± 0.15 ps (see Fig. S6 in the SI) No signal offset at long delay times was observed. However, only limited information on the electronic states involved in the deactivation could be deduced from these data, because the intermediate and final states of the deactivation cascade could not be identified with the 800 nm probe. We therefore switched to X-ray probe schemes to unravel the photophysics of the molecule.

### 2.1 TR-XPS

**Fig. 2:**
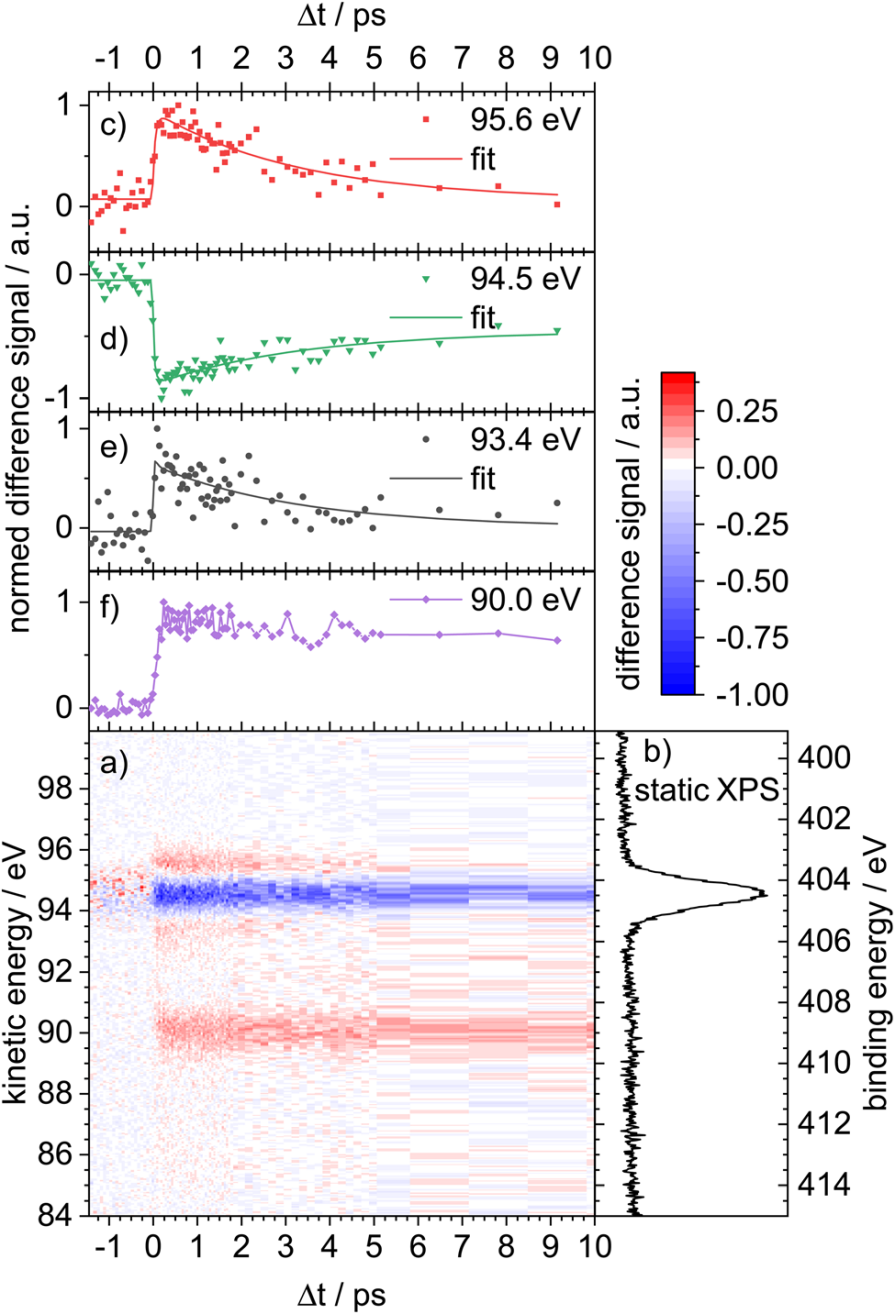
(a) Time-resolved X-ray photoelectron spectrum of phenanthridine displayed as a 2D map (difference spectrum), using *hν*_pump_ = 4.63 eV (268 nm) and *hν*_probe_ = 499 eV. The bands in the map correspond to N 1s ionization. (b) Static XPS spectrum of phenanthridine (UV off). (c)–(f) Delay traces for selected kinetic energies (eKE) in the 2D map. A ground state bleach signal is visible around eKE = 94.5 eV (d). Two weaker transients around 93.4 eV (e) and 95.6 eV (c) are assigned to the excited-state dynamics of phenanthridine. The absorption band at eKE = 90 eV (f) shows no decay and is due to two-photon ionization of phenanthridine with subsequent N 1s ionization of the cation.

Fig. 2a shows the TR-XPS spectrogram obtained by ionization of a N 1s electron in the probe step with *hν*_probe_ = 499 eV (blue arrow on the right hand side of Fig. 1). The 2D map displays the intensity of the difference photoelectron signal as a function of both electron kinetic energy (eKE, left ordinate) and binding energy *E*_B_ (with *E*_B_ = *hν*_probe_ − eKE, right ordinate), and the pump–probe delay Δ*t* on the abscissa. The time-independent background signal (pump laser off) due to the XPS probe alone has been subtracted. Red bands in the 2D map indicate a signal increase upon interaction with the pump laser, while the blue color indicates a signal that decreases with respect to the background signal after pump excitation. The most pronounced feature is associated with a depleted photoelectron signal centered at eKE = 94.5 eV. It is assigned to a ground state bleach (GSB). From the static XPS in Fig. 2b, a N 1s ionization energy (or *E*_B_) of 404.5 eV is deduced, which is in line with the value for related molecules like pyridine (404.82 eV).^36^ On the time scale of the experiment, the GSB does not fully recover. In addition, three photoelectron bands are recognized after *t*_0_ at *E*_B_ = 409.0 eV, 405.6 eV and 403.4 eV (eKE = 90.0 eV, 93.4 eV and 95.6 eV). For these three bands and for the GSB, the corresponding delay traces are given in Fig. 2c–f. The one at eKE = 90 eV (Fig. 2f) shows no decay and the chemical shift Δ*E* = 5 eV is too large to be reasonably assigned to an excited electronic state of 1. In addition, the signal intensity depended sensitively on the pump laser pulse energy which was varied between 10 and 30 μJ. A spectrum recorded at 20 μJ is given for comparison in Fig. S7 the SI. All bands are present, but the analysis is hampered by the low signal/noise ratio. Therefore, we assign the feature at eKE = 90 eV to the phenanthridine cation 1^+^, formed by a two-photon ionization in the pump step. Unsurprisingly, N 1s ionization of 1^+^ is associated with higher binding energies, because more energy is required to remove a 1s electron from the cation. The latter two absorption bands, however, are assigned to the intermediate state dynamics of 1. Qualitatively, the band at eKE = 93.4 eV (Fig. 2e) appears directly after excitation and decays rapidly. The band at eKE = 95.6 eV (Fig. 2c) seems to appear slightly later in time and exhibits a pronounced component with a longer lifetime. A global fit using the model outlined in Section 2 (see also Section S10 for details) reveals two time constants, *τ*_1_ ≤ 100 fs and *τ*_2_ = 3.3 ± 0.8 ps. Constant *τ*_1_ seems to govern the decay of the 93.4 eV band (trace e) and the risetime of the 95.6 eV band (trace c), while *τ*_2_ describes the decay of the 95.6 eV band and the recovery time of the GSB (trace d). The determination of more accurate values is impeded by the signal-to-noise (S/N) ratio of the data.

To fully understand the processes in the S_0_ state, TR-XPS spectra were recorded up to 500 ps, using the same experimental conditions. As can be seen from Fig. S8, the signal attributed to the GSB starts to increase again after 10 ps. A further transient appears with time, broadens and starts to shift to lower kinetic energies. This band has a temporal profile that is similar to the GSB. In isolated molecules, such a change indicates either dissociation or isomerization in the S_0_ state and formation of a new species with a slightly lower *E*_B_ of 406.4 eV. Interestingly, this value matches the *E*_B_ of HCN of 406.69 eV quite well.^37^ We therefore tentatively assign this band to the formation of a HCN photoproduct from the electronic ground state of the neutral. The rise time on the order of several 100 ps is commensurate with a unimolecular dissociation that can be described by statistical rate theory. At least in PANH cations this process (HCN loss) is well documented.^38^ RRKM calculations were not carried out, because reasonably accurate barriers are necessary, which requires a comprehensive calculation of all possible reaction pathways leading to HCN loss.

### 2.2 TR-XAS

**Fig. 3:**
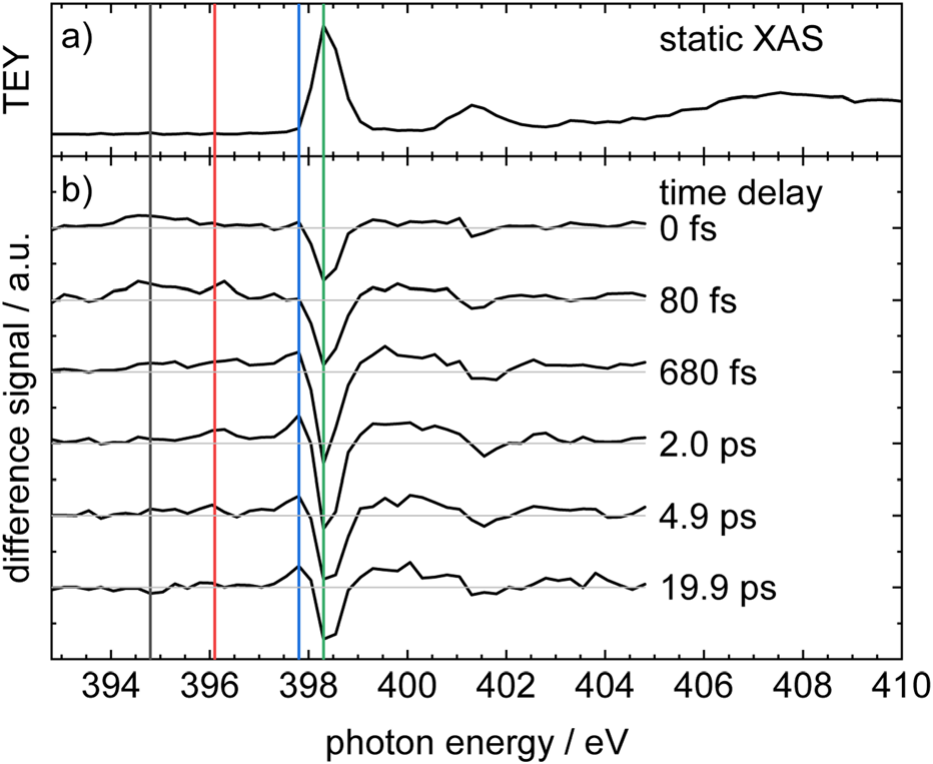
Experimental X-ray absorption spectra of phenanthridine measured as total electron yield (TEY): (a) static XAS and (b) ΔXAS at different pump–probe delay times. Vertical lines indicate probe photon energies where delay traces were recorded.

To complement TR-XPS, time-resolved X-ray absorption spectra were recorded at the N 1s edge starting from UV valence excited 1 (*λ*_pump_ = 268 nm). As illustrated on the right hand side of Fig. 1, the N 1s electron can be promoted into the half-filled (green dashed and dotted lines) or empty orbitals (green dash-dotted line) of the optically excited molecule, *i.e.*, transitions such as 1s → n and 1s → π occur. The static X-ray absorption spectrum (Fig. 3a) shows an intense band at 398.3 eV that is due to excitation of the N 1s electron into the π* orbital (LUMO). A second band at 401.3 eV is due to a transition into a higher unoccupied level (*vide infra*). In accordance with the XPS data above, the ionization limit is reached between 404 eV and 405 eV and leads to an increase of the XAS signal.

**Fig. 4:**
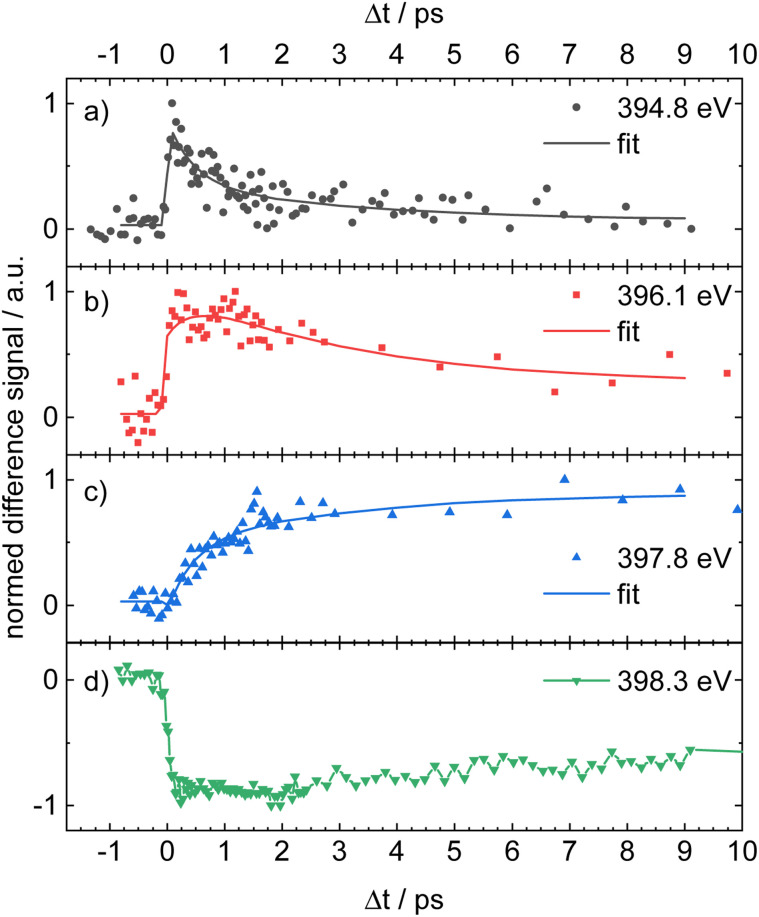
Time delay traces of the XAS experiments. The color code represents the lines given in Fig. 3. The band at 394.8 eV (a), (black line, circles) shows a rapid decay, whereas the band at 396.1 eV (b), (red line, squares) decays more slowly. The band at 397.8 eV (c), (blue line, triangles) shows a monoexponential rise and is assigned to hot ground state absorption. The bottom trace (d), (green line, reverse triangle) finally depicts the GSB. The time constants *τ*_1_ = 0.45 ± 0.18 ps and *τ*_2_ = 3.1 ± 1.8 ps were determined in a global fit.

Fig. 3b shows ΔXAS recorded at various time delays, with ΔXAS = XAS(*t*) − XAS(probe only). At several probe photon energies, marked as colored lines in the experimental spectrum, the signal was subsequently measured as a function of time delay. The time-dependence of these traces is shown in Fig. 4. An initial qualitative discussion will be based on Fig. 3. Exciting 1 at 268 nm results in a depletion of population in the electronic ground state, and subsequently a time-dependent bleach of the 1s → LUMO signal (green line at 398.3 eV). A further bleach is apparent for the second band at 401.3 eV. As it is also due to depopulation of the S_0_ state, its time-dependence is very similar to the 1s → LUMO signal. Several transient absorption (TA) features appear towards lower energies. The lowest energy transient feature at 394.8 eV (black line) appears immediately after the pump pulse and is therefore assigned to TA from an electronic state that is either populated upon excitation or very rapidly thereafter. It is shifted by *ca.* −3.5 eV relative to the 1s → LUMO ground state absorption and reaches its highest intensity in the 80 fs trace, before it decreases again. The second band at 396.1 eV (red line) appears slightly later and decreases on a ps-time scale. It is therefore associated with a transiently populated electronic state. Finally, the band at 397.8 eV (blue line) starts to appear between 80 fs and 680 fs and increases on a ps-time scale. As it is very close in energy to the GSB, we assign this band to absorption of vibrationally excited molecules in the S_0_ state. Alternatives to hot ground state formation would be an excited state dissociation, which should be visible in the TR-XPS at short delay times, and radiative decay, which would occur on a longer time scale. Thus, after non-radiative deactivation, energy is instead redistributed among the vibrational degrees of freedom, forming a hot ground state. This band assignment is confirmed by computations, as described below. There are several further TA bands in between the two GSB features at 398.3 eV and 401.3 eV. At the high energy side of the major band (398.3 eV) we also expect hot ground state absorption. However, there is an overlap with transitions terminating in higher unoccupied orbitals, so it becomes increasingly difficult to disentangle the various contributions. Therefore, these features are not further discussed.

In addition to the transitions in neutral phenanthridine, we also expect a transition in the phenanthridine ion 1^+^, corresponding to the band at *E*_B_ = 409 eV in the XPS, which was assigned to two-photon ionization of 1. The XAS of 1^+^ has not been studied, but for some small molecules, including N_2_^+^ XAS of the cations are available.^39^ In that case, the 1s → SOMO peak of the ion appears at an energy that corresponds to the binding energy in the XPS minus the IE of the neutral. Inserting the values for 1, we obtain 396.2 eV for the likely transition in 1^+^. This value is very close to the band at 396.1 eV. Thus, a contribution from 1^+^ can be expected for the latter band.

Fig. 4 shows the time delay traces obtained for absorption energies of 394.8 eV (a), 396.1 eV (b) and 397.8 eV (c), as well as the GSB at 398.3 eV (d), corresponding to the bands labeled with lines in black, red, blue and green in Fig. 3. The top trace (Fig. 4a) shows a rapid rise within the instrument response function (irf) of *ca.* 100 fs, followed by a fast decay. In contrast, the band at 397.8 eV (Fig. 4c) shows a monoexponential rise on the ps-scale, which matches the formation of a hot ground state. The GSB itself (Fig. 4d) shows a slight recovery, but increases again after *ca.* 10 ps, *cf.* Fig. S8. The band in Fig. 4b, corresponding to a probe photon energy of 396.1 eV (red), is more difficult to interpret and has an unusual appearance. After a rapid increase, the absorption signal decreases in a complicated fashion and seems to show multiple components. The signal also does not decrease to zero, but rather reaches a finite value. As outlined above, the band is most likely perturbed by the time-independent absorption of 1^+^. Both contributions are independent, because they originate from different processes. Their presence nevertheless renders the determination of time constants more difficult. We therefore assume that the signal is composed of a quickly rising contribution of 1^+^ that remains constant on the time scale of the experiment and a contribution from 1 with a few ps decay.

The solid lines in Fig. 4a–c represent a global fit of all three traces, again employing the previously described model, which yields two time constants, *τ*_1_ = 0.45 ± 0.18 ps and *τ*_2_ = 3.1 ± 1.8 ps.

### 2.3 Nonadiabatic dynamics

To gain further insight into the electronic states involved in the photodynamics, trajectory surface hopping simulations over 1 ps were performed on a set of 50 trajectories of 1 launched from their brightest excited states: 26 were started from the S_2_ state, while the other 24 were started from the S_3_ state.

**Fig. 5:**
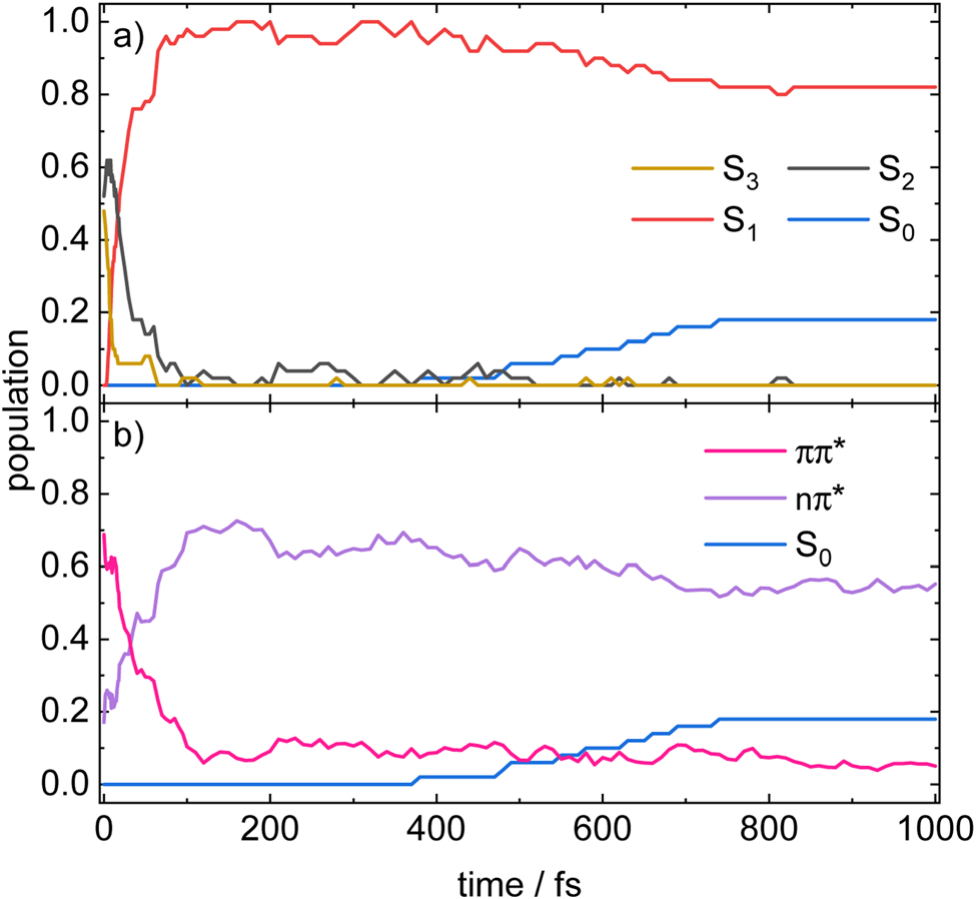
Time evolution of the populations in the (a) adiabatic states and (b) diabatic states, averaged over 50 trajectories.

The time evolution of the populations in the adiabatic picture is shown in Fig. 5a. The excited state deactivation of 1 from S_2_ or S_3_ to S_1_ takes place within the first *ca.* 60 fs. The population transfer from S_3_ to S_2_ is essentially completed within 10 fs, while the decay from S_2_ to S_1_ is slower. All trajectories remain in this lowest excited singlet state from roughly 100 fs on; occasionally, some trajectories hop between S_1_ and S_2_ (and S_3_). After about 500 to 700 fs, nine trajectories have returned to the electronic ground state. However, the switch to the electronic ground state is not well represented in the framework of TDDFT, because the multi-reference character of S_0_ in the region of the conical intersection between S_1_ and S_0_ is not taken into account.

To analyse the character of the electronic states during deactivation, a diabatisation along the nuclear trajectories was performed as described in Section S6 of the SI. The results of the diabatisation are depicted in Fig. 5b. The initial ensemble as excited into the S_3_ and S_2_ state has primarily ππ* character. The S_2_ state, although formally an nπ* state, gains some ππ* character due to vibronic coupling. The initial rise in the S_2_ population within the first 10 fs correlates with the concomitant drop in the ππ* population, indicating that molecules excited to S_3_ convert into an S_2_ of predominantly nπ* character, whereas those directly excited to S_2_ retain their ππ* character. A short section of almost constant population observed for both S_2_ and ππ* between 5 and 15 fs signifies that the diabatic character of S_2_ is preserved.

Subsequently, S_2_ decays faster than the ππ* population, showing that the nascent S_1_ state carries a substantial ππ* contribution. Once S_2_ is depleted, the S_1_ character continues to change from ππ* to nπ* character for some 100 fs. *I.e.* once the trajectory reaches S_1_, it experiences an intrastate shift from ππ* to nπ* character. After ≈500 fs return to the electronic ground state sets in. To obtain a deeper insight into the dependence of the electronic structure of 1 on the nuclear geometry, the potential energy surfaces were scanned for selected vibrational modes. The normal modes that dominate the relaxation dynamics starting from the S_3_ state were identified by transformation of the time-dependent cartesian coordinates into time-dependent normal coordinates by means of a projection of the trajectories onto the normal modes of 1 in the FC structure.^40^ For more details, see Section S7 of the SI. The investigation revealed that the vibronic coupling is mainly mediated by three in-plane bending modes (see Fig. S10) along which the potential energy surfaces exhibit pronounced crossings, as can be seen from Fig. S11.

To support the interpretation of the experimental data, the X-ray absorption spectra were simulated based on EOM-CCSD computations that include core hole excitations and, to further aim for simulations of time-resolved XAS, employing the less expensive CASPT2/RASPT2 method (abbreviated as RASPT2 in the following) for which the chosen active space is depicted in Fig. S2. This calculation type produces a set of orbitals by unitary transformation of the molecular orbitals, resulting in the so-called natural orbitals (NOs) which do not have physically meaningful energies, and are therefore arbitrarily ordered. The orbital labels used in this work, along with their corresponding characters, are provided in Table 2, (a). The vertical excitation energies *E*_exc_ and the oscillator strengths *f* given in Table 2, (b) are in good agreement with the TDDFT results (*cf.*Table 1, (a)). When comparing computed with experimental intensities, their method-dependence should nevertheless be taken into account. The excited state composition at CASPT2 level of theory (Table 2, (b)) is in line with the TDDFT calculation regarding the S_1_ and S_2_ states, whereas the mixed character of S_3_ is not well reproduced. However, this state is populated only for a very short time, so its contribution to the TR-XAS simulated at RASPT2 level is expected to be of minor importance.

(a) Labels and characters of the natural orbitals. (b) Vertical excitation energies *E*_exc_ and oscillator strengths *f* for the three lowest excited singlet states and the corresponding dominant transition characters along with the state compositions computed at the Franck–Condon geometry. The data were computed at SA4-RMS-CASPT2(6,5)/cc-pVDZ level of theory (for more details, see Section 5.2)

**Table:** 

(a)	
Label	Character
LUNO+1	π*
LUNO	π*
HONO	n
HONO−1	π
HONO−2	π

**Table:** 

(b)			
State	Composition	E_exc_/eV	*f*
S_1_ (ππ*)	54% HONO−1 → LUNO	4.09	0.0203
	27% HONO−2 → LUNO+1		
S_2_ (nπ*)	90% HONO → LUNO	4.54	0.0066
S_3_ (ππ*)	91% HONO−1 → LUNO+1	4.67	0.6965

**Fig. 6:**
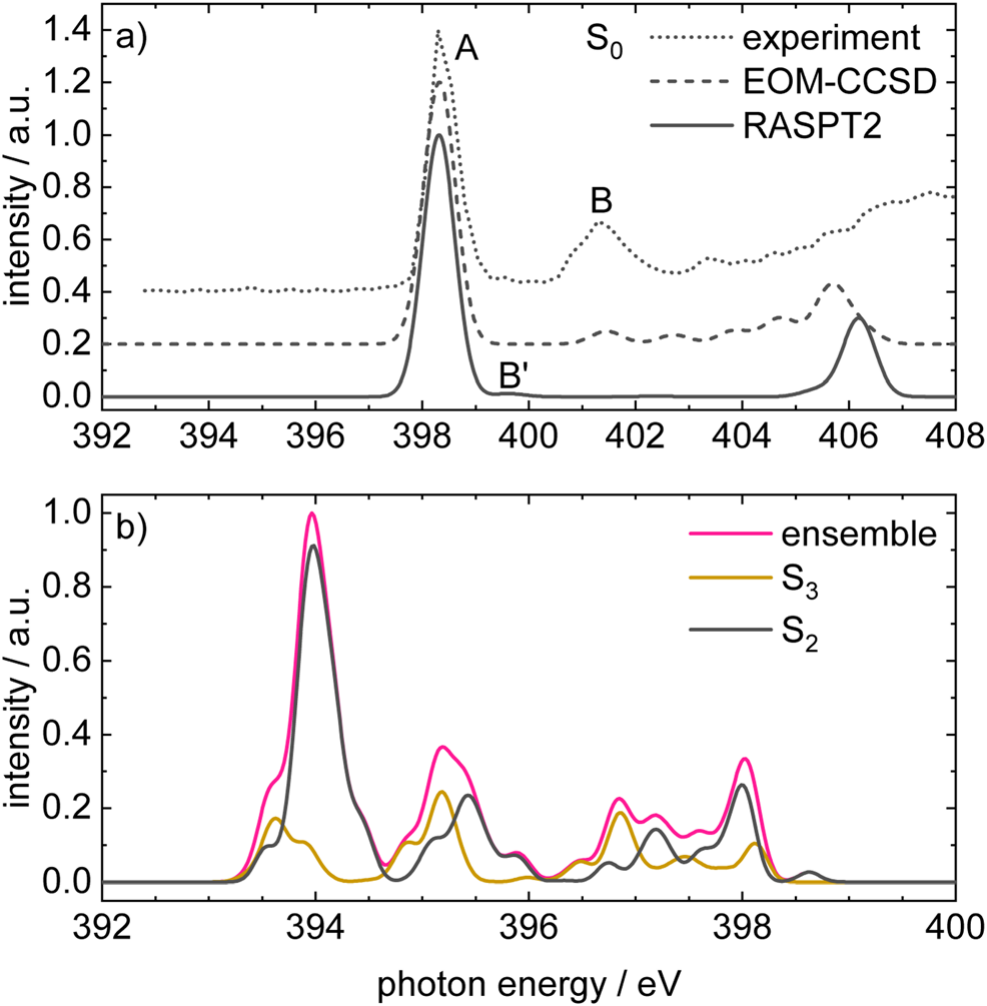
(a) Comparison of the experimental static XAS with the simulated XAS for the electronic ground state computed using EOM-CCSD and RASPT2. All calculations were carried out at the equilibrium structure of 1 and the simulated stick spectra were broadened with a Gaussian profile with the width of *σ* = 0.3 eV. The EOM-CCSD (dashed) and RASPT2 (solid) spectra were shifted by −4.0 eV and −3.1 eV, respectively. (b) Simulated XAS of the ensemble used in the nonadiabatic dynamics, computed at the RASPT2 level. The magenta trace is the total ensemble-averaged XAS at time zero, while the yellow and black traces show the sub-ensemble contributions from molecules initially promoted to S_3_ or S_2_, respectively. All stick spectra were shifted by −3.1 eV and broadened with a Gaussian of width *σ* = 0.1 eV. The smaller Gaussian width in panel (b) reflects the fact that the ensemble's intrinsic distribution of molecular geometries already contributes to significant spectral broadening.

Fig. 6a compares the simulated X-ray absorption spectra at the equilibrium structure of 1 in the electronic ground state computed at EOM-CCSD (dashed line) and RASPT2 (solid line) level of theory with the experimental spectrum (dotted line). The EOM-CCSD simulation was shifted by −4.0 eV so that the 1s → LUMO absorption band (A) coincides with the most intense band in the experimental spectrum. The computed spectrum is in good agreement with the experimental one for the signal at *ca.* 401.3 eV (B), which can be assigned to the mixed transition involving 1s → LUMO+1 (55%) and 1s → LUMO+2 (22%) orbital excitations. The RASPT2 simulation was shifted by −3.1 eV analogously. Due to the absence of the LUMO+2 in its active space, the band B is not well reproduced in the RASPT2 spectrum and appears at 399.6 eV (B′).

Fig. 6b displays the simulated XAS of the ensemble used in the nonadiabatic dynamics, computed at the RASPT2 level. The magenta trace is the total ensemble-averaged XAS at time zero, while the yellow and black traces show the sub-ensemble contributions from molecules initially promoted to S_3_ or S_2_, respectively. The band at 394.0 eV with the high-energy shoulder at 394.5 eV is attributed to the excited-state absorption (ESA) from S_2_, because the intensity of the excitation from S_3_ is very low at this energy window. The band around 395.2 eV results from absorptions of both S_2_ and S_3_ into higher core-excited states. As the excited population at time zero has mainly ππ* character the signals result from a 1s → π transition to a large extent, although a non-negligible contribution from 1s → n is present, *cf.*Fig. 5b.

**Fig. 7:**
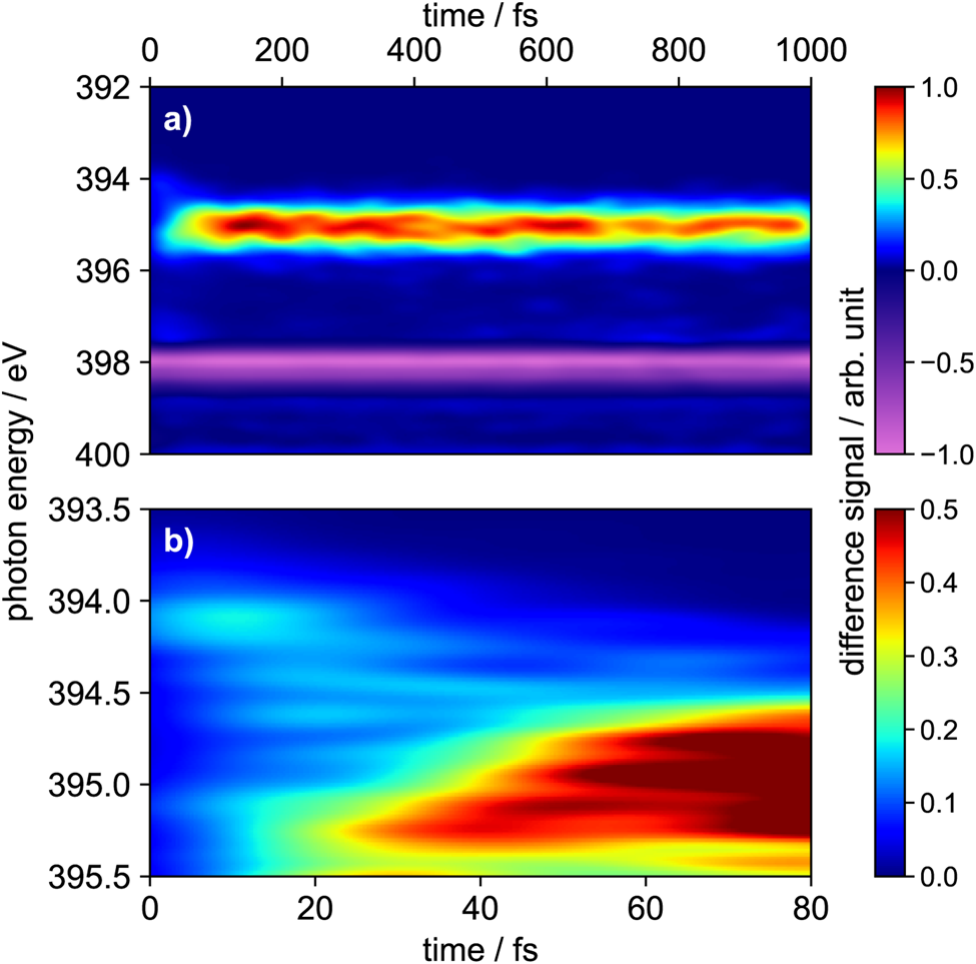
(a) Simulated transient X-ray absorption spectrum of 1 computed using the RASPT2 method. The ensemble ground state spectrum was broadened with a Gaussian profile with the width of *σ*_exc_ = 0.1 eV, the transient TR-XAS was broadened with a two-dimensional Gaussian profile with the standard deviations of *σ*_exc_ = 0.1 eV and *σ*_t_ = 20 fs. (b) Close-up of the of the first 80 fs between 393.5–395.5 eV, where the spectrum was broadened with a two-dimensional Gaussian profile with *σ*_exc_ = 0.05 eV and *σ*_t_ = 10 fs.

The time-resolved XAS shown in Fig. 7a is computed by subtracting the ground state XAS obtained at time zero from the XAS computed along the surface hopping trajectories (broadened with a two-dimensional Gaussian profile with *σ*_exc_ = 0.1 eV and *σ*_t_ = 20 fs). All computations are performed at RASPT2 level. Fig. 7b depicts the relevant time and energy region for the ultrafast decay of the S_2_ state, where narrower two-dimensional Gaussian profiles were used. Again, the energies have been shifted by −3.1 eV to match the experimental static XAS. For enhanced visibility the individual contributions of the transient population in the S_0_, S_1_, S_2_ and S_3_ state are given separately in Fig. S12.

The light blue feature at around 394.1 eV visible in Fig. 7b that appears already at time zero arises from ESA of the sub-ensemble initially excited to S_2_. This can be inferred from the ensemble XAS in Fig. 6b and the S_2_ state-selective TR-XAS in Fig. S12. Between 5 and 10 fs signals at 394.4 eV and 394.6 eV (two light blue stripes) grow in alongside a further intensification of the 394.1 eV band, reflecting the ultrafast decay of S_3_ and concurrent rise of the S_2_ population (*cf.*Fig. 5). After about 20 fs, these S_2_-related features weaken while a new broad signal between 394.6 and 395.3 eV appears and intensifies, which is caused by ESA from S_1_ as recognised from Fig. S12. Finally, as visible in trace (a) beyond *ca.* 600 fs, the S_1_ signature decays and a feature at 397.3 eV emerges, marking the recovery of the hot electronic ground state, accompanied by a hot ground state feature. This feature is best recognised in the S_0_ state-selective TR-XAS shown in Fig. S12.

Fig. 7 supports the following assignments for the experimental time delay traces in Fig. 4:

• The trace at 394.8 eV (black line in Fig. 4a) follows the energy and kinetics of the S_2_ ESA in the simulated TR-XAS.

• The trace at 397.8 eV (blue line in Fig. 4c) coincides with the hot ground-state XAS.

• The trace at 398.3 eV (green line in Fig. 4d) matches the GSB.

All three features are therefore assigned to ESA of S_2_, hot ground state XAS, and GSB, respectively. The trace at 396.1 eV (red line in Fig. 4b) follows the temporal profile of simulated S_1_ ESA but is shifted by about 1.1 eV. This offset is attributed to the limited active space in our RASPT2 calculations; nonetheless, the qualitative agreement remains valid (see Section S9 in the SI). As noted above, the experimental band also contains contributions from 1^+^.

**Fig. 8:**
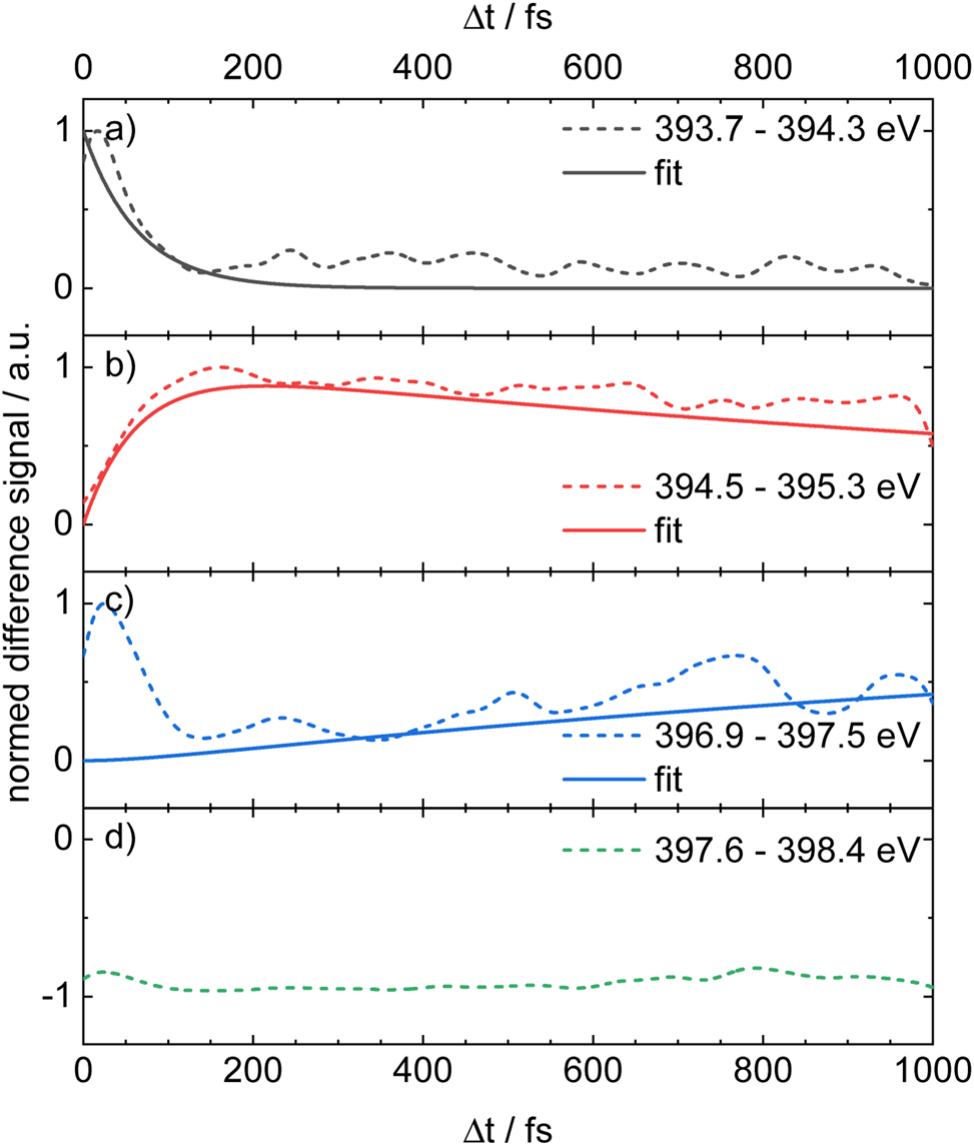
Simulated time delay traces for selected bands in the computed transient X-ray absorption spectrum (Fig. 7) using a temporal broadening of 20 fs. Traces (a)–(d) correspond to the transients displayed in Fig. 4.

For a direct comparison with the experiment, the simulated counterparts of the four experimental time delay traces in Fig. 4 are plotted in Fig. 8 as dashed lines. Overall, the simulation reproduces the main temporal features, with the exception of the blue trace in panel (c), which exhibits an elevated signal at time zero. We attribute this initial offset to ESA from S_2_ and S_3_ into highly excited core-level states that are likely poorly described by the limited active space in our RASPT2 calculations and therefore deviate from the experiment. The transients in panels (a), (b) and (c) were fitted with a sequential two-step deactivation model (solid lines in Fig. 8), yielding time constants of *τ*_1_ = 63 fs and *τ*_2_ = 1.7 ps. These values are in reasonable agreement with the experimental lifetimes.

## 3 Discussion

The experimental and computational data presented above provide a detailed insight into the photodynamics of 1. In this section, we will put the various observations in context and derive a consistent interpretation of the data. At 268 nm, the molecule is excited into the 2ππ* state. Previous REMPI spectra (Fig. S4) showed an intense band that is broad and unstructured.^32^ Although this study suggests that this band corresponds to the S_3_ ← S_0_ transition we prefer to describe it as a 2ππ* ← S_0_ transition, because the 2ππ* character dominates the absorption of the ensemble.

Three different probe methods were employed, 800 nm multiphoton ionization using a femtosecond laser (Fig. S6, time-resolution ≈150 fs), TR-XPS (Fig. 2) and TR-XAS (Fig. 3) with X-ray FEL radiation (time-resolution ≈100 fs, see below). In all three approaches, the time-dependence of the signal was fitted by a biexponential function. The fitting procedure is described in Section S10 of the SI, while the time constants obtained in the experiments are summarized in Table 3. They deviate somewhat quantitatively, in particular *τ*_1_ obtained by TR-XPS is smaller, possibly due to overpumping in the FEL experiments or due to an inferior signal/noise ratio, but agree qualitatively quite well. The first one, *τ*_1_, is on the fs-scale and lies around 300 fs, while *τ*_2_ is around 3 to 4 ps. Thus, the data agree with a two-step deactivation. In contrast, theory suggests a three-step process, with two sub-100 fs time constants and a third one on the ps-time scale. We therefore introduced a third time constant in the fit to the experiments, but this did not improve the fit further.

**Table 3:** Time constants determined in the various experiments

Method	TR-PES	TR-XPS	TR-XAS
*τ*_1_/ps	0.27 ± 0.05	≤ 0.1	0.45 ± 0.18
*τ*_2_/ps	4.3 ± 1.3	3.3 ± 0.8	3.1 ± 1.8

It is the main goal of the current study to unravel the detailed dynamics of the deactivation cascade and correlate the time constants with specific processes. The best information to this end is available from the TR-XAS. Here, a band shifted by 3.5 eV to lower energies (black line in Fig. 3b) appears right after the excitation and might at first glance be assigned to TA from the S_3_ 2ππ* state. However, the surface hopping simulations suggest very fast sub-100 fs time constants for both the S_3_ → S_2_ and S_2_ → S_1_ deactivation (*cf.*Fig. 5). As we only extract one fast time constant from the experimental data, we conclude that it corresponds to the S_2_ → S_1_ transition, because the calculated oscillator strength for the ESA of S_2_ is significantly larger than that for S_3_. Therefore, the contribution from S_3_ (*cf.*Fig. 6b) is not visible in the experimental spectrum. The second time constant is then associated with return of population to the electronic ground state. This is evidenced by the GSB signal in the TR-XPS at *E*_B_ = 404.5 eV (eKE = 94.5 eV), *cf.*Fig. 2d, as well as the decay of the transient absorptions at 396.1 eV (red line in Fig. 4b) and the rise at 397.8 eV (blue line in Fig. 4c, formation of hot ground state). Thus, *τ*_2_ is assigned to an internal conversion (IC) from the S_1_ state to the electronic ground state. The experimental evidence is in line with the simulated excited state spectra (Fig. 7 and 8) and the computed time constants of 63 fs and 1.7 ps. As noted above, the XAS band at 396.1 eV likely contains a contribution from the time-independent absorption of the cation (Fig. 4b). This increases the error in the determination of the time constants.

The bands in the TR-XPS spectrum (Fig. 2) can in principle be assigned based on the local charges on the N-atom. A population analysis based on Löwdin charges revealed the highest positive charge density on the nitrogen atom in the nπ* state. This results in a higher *E*_B_ (and lower eKE) for ionization from the nπ* state and suggests the band *E*_B_ = 405.6 eV (eKE = 93.4 eV, Fig. 2e, black line) to be associated with this processes. However, as both S_2_ and S_1_ have a mixed ππ*/nπ* character that evolves during deactivation, an assignment based on local charges only seems inadequate.

The surface hopping computations also provide information on the electronic character of the states. Over time, the initially dominant ππ* character in the S_1_ state changes to nπ* character. More precisely, the sub-ensemble originally excited to the S_2_ state follows the deactivation cascade 2ππ* → 1ππ* → nπ* → 
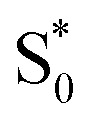
, while the sub-ensemble originally excited to the S_3_ state decays according to 2ππ* → nπ* → 1ππ* → nπ* → 
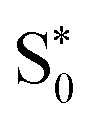
. Note that the fast time constants are not commensurate with intersystem crossing, which is therefore not further considered.

Once back in the hot ground state 
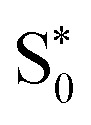
, 1 dissociates with a time constant of several hundreds of ps. As concluded from the TR-XPS data at longer delay times, dissociation is likely associated with HCN loss, *cf.* Fig. S8. Dissociation in PAH is often associated with loss of H_2_ or C_2_H_2_ and can usually be described in a statistical model. In PANH cations, loss of HCN constitutes an alternative to acetylene loss reaction and is often dominant,^38^ its appearance in 1 would therefore not surprising. This possible loss of HCN in neutral PANH might also be of relevance for astrochemistry.

The data obtained here apply to deep UV excitation, but also permit to expand the interpretation to lower energies, *i.e.*, the ^1^(1ππ*) state. As shown in Fig. S5 in the SI, a ns-lifetime has been found for S_1_ 0^0^ band, while decay times between roughly 10 and 100 ps have been observed for the well resolved excited vibronic bands. A signal offset at long delay times shows that the final state can be efficiently ionized. We therefore assign the decay to a ^1^(1ππ*) → ^1^(nπ*) internal conversion. At low energies, the ^1^(nπ*) is long-lived. However, when the ^1^(2ππ*) state is accessed, the dynamics accelerate dramatically, following the mechanism outlined above.

## 4 Conclusions

In this work, we studied the excited state deactivation of the polycyclic aromatic nitrogen-containing hydrocarbon phenanthridine after UV excitation at 268 nm into the bright 2ππ* state. The analysis was based on time-resolved X-ray photoelectron and X-ray absorption spectra at the nitrogen 1s edge as well as valence photoionisation with *λ*_probe_ = 800 nm, along with trajectory surface hopping computations and time-resolved X-ray absorption simulations. The experimental data suggested two time constants of *ca.* 0.3 ps and 3 ps. Since the trajectory simulations predicted a very fast sub-10 fs time constant for the S_3_ → S_2_ process, we conclude that it is too fast to be time resolved in the experiments. The S_2_ → S_1_ transition appears in the TR-XAS as the lowest energy transient feature that rises immediately after the pump pulse and shows a rapid decay. All methods confirmed the return of the population to the electronic ground state, so the second time constant was readily assigned to the internal conversion from S_1_ to S_0_. This process is reflected in the TR-XAS by a transient absorption signal that decays more slowly along with a reduction of the ground state bleach. In addition, the formation of vibrationally excited molecules in the electronic ground state was observed. The TR-XPS data confirm the mechanism outlined above, but also provide additional information. They indicate the presence of a contribution from 1^+^ that shows no fast dynamics and is difficult to isolate in the TR-XAS. Furthermore, on a time scale of several hundreds of ps, a unimolecular dissociation of 1 is apparent in the TR-XPS, possibly associated with loss of HCN.

Data using *λ*_probe_ = 800 nm probably yield more accurate time constants, but cannot identify the intermediate states in the deactivation process, while picosecond time-resolved experiments allow to extend the interpretation to lower excitation energies.

Theory and experiment together conclude that the ultrafast excited state deactivation of phenanthridine takes place according to 2ππ* → 1ππ* → nπ* → 
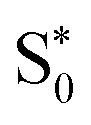
 for the sub-ensemble originally excited to the S_2_ state and according to 2ππ* → nπ* → 1ππ* → nπ* → 
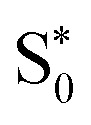
 for the sub-ensemble originally excited to the S_3_ state. The interpretation was supported by investigating the potential energy surfaces of the lowest three excited singlet states along the dominant normal modes responsible for the relaxation dynamics (in-plane bendings). They exhibit pronounced crossings, allowing for an ultrafast internal conversion in phenanthridine. Three general conclusions are derived from the present work: (a) as expected, the photochemistry of phenanthridine is significantly more complex than in the pure hydrocarbon PAH and involves nπ* states in addition to ππ* states. (b) Based on previously observed similar electronic spectra of other aza-phenanthrene isomers,^32^ a related deactivation mechanism is expected to govern their photochemistry. Finally, (c) time-resolved X-ray spectroscopy at the N 1s edge is an excellent tool to unravel the detailed photochemistry of N-heterocycles, in particular when combined with high-level computations that properly represent the time-resolved X-ray absorption spectrum.

## 5 Methods

### 5.1 Experimental

The experiments were performed at the soft X-ray beamline Athos of the SwissFEL free-electron laser,^41^ using the Maloja endstation. The experiment operated at a repetition rate of 100 Hz. The 30–40 fs pump pulses centered at 268 nm (FWHM ≈ 4 nm) were derived from the third harmonics of a Ti:Sa laser system and the pulse energy was varied between 10 and 200 μJ by tuning a waveplate. In the experiments described above, an energy of 30 μJ was employed. The instrument response function was around 100 fs. The FEL is operated in self-amplified spontaneous emission (SASE) mode. Using the Athos monochromator, a photon bandwidth of 0.4 eV is cut out of the original 1% energy bandwidth SASE pulses. This value also determines the energy resolution of the spectra to Δ*E* ≈ 0.1%. The undulators and the monochromator were aligned to a fixed photon energy of 499 eV for the TR-XPS experiments, while for the TR-XAS experiments the photon energy of the FEL was tuned between 393 eV and 405 eV. The X-ray pulse energy at the sample was around 50 μJ, a focus size of 100 × 100 μm is estimated, assuming a Gaussian beam profile. The X-rays and the optical laser beams were overlapped by a drilled mirror. For every fifth FEL pulse, the pump laser was delayed and the probe-only signal was recorded for background subtraction. The FEL pulse energy was constantly monitored by detecting the Ar ion signal in a time-of-flight mass spectrometer for every pulse, permitting to normalize all data to the FEL pulse energy. The time intervals between the points varied between 45 fs close to the pump–probe overlap and up to 1.3 ps far away from it, the points being measured randomly. Typically, in each run 2000 pulses were averaged per data point and four (sixteen) runs were averaged for the XAS (XPS) spectra shown above.

A Specs PHOIBOS 150EP hemispherical analyzer was employed for electron detection. For TR-XPS, it was operated at a pass energy of *E*_P_ = 240 eV (photoelectrons were accelerated appropriately) and calibrated by ionizing N_2_ with the FEL. For TR-XAS, conditions in the analyzer were changed to detection of Auger electrons and X-ray absorption spectra of N_2_ were recorded for photon energy calibration.

The sample was obtained from Sigma-Aldrich and used without purification. It was slightly heated in an oven source kept at 60 to 65 °C, the pressure in the experimental chamber was kept at *p* ≈ 1 × 10^−6^ mbar.

### 5.2 Theory

Computations on 1 were carried out using time-dependent density functional theory (TD-DFT) employing a long-range corrected hybrid functional ωB97X-D^42^ combined with the def2-SV(P)^43^ basis set, as implemented in the Q-Chem software package.^44^ For trajectory-based nonadiabatic dynamics simulations with four electronic states, Tully's fewest-switches surface hopping^45^ procedure was applied using the ωB97X-D/def2-SV(P) electronic structure method.^46,47^ For more details, see Section S5 of the SI. A total of 50 initial structures were generated by sampling a harmonic canonical Wigner distribution^48,49^ at 330 K and were subsequently relaxed by propagating for 100 fs in the electronic ground state without any thermostats. The relaxed geometries were launched from the brightest electronic state and the trajectories were propagated for 1 ps with a time step of 0.25 fs. For simulating the X-ray absorption spectra, the equation-of-motion coupled-cluster formalism with singles and doubles (EOM-CCSD)^50^ combined with the cc-pVDZ^51^ basis set was employed. The core-level states were included by the core–valence separation (CVS) scheme.^52^ Additionally, the state-averaged complete active space self-consistent field method^53^ (SA-CASSCF) including the first four singlet states using the cc-pVDZ basis set was applied, as implemented in the OpenMolcas v23.02 software package.^54^ Second-order multiconfigurational perturbation theory was added employing the rotated multistate approach (SA-RMS-CASPT2).^55^ As an active space, six electrons in five orbitals were selected, labeled as SA4-RMS-CASPT2(6,5). This procedure was extended to the state-averaged restricted active space self-consistent field method,^56^ followed by multiconfigurational perturbation treatment (RMS-RASPT2).^55^ The nitrogen 1s orbital was chosen as the RAS1 space and the RAS2 space included six electrons in five orbitals as before. Hence, the resulting restricted active space consisted of eight electrons in six orbitals, labeled SA-RMS-RASPT2(8,6). Because state mixing of the SA-CASSCF wavefunction becomes significant upon geometric distortion, we included the first six singlet states in the SA-CASSCF to ensure correct state ordering for both ensemble XAS and TR-XAS. Valence excited states calculated using SA6-RMS-CASPT2(6,5) then served as reference configurations for the promotion of exactly one electron from the nitrogen core orbital forced within the subsequent SA10-RMS-RASPT2(8,6) calculation considering the lowest ten X-ray excited states. For reasons of computational efficiency, the spectrum along the trajectories was determined every femtosecond within the first 20 fs, then every 5 fs up to 100 fs and then every 10 fs.

## Author contributions

Dorothee Schaffner: lead (experiment & data analysis), writing (original draft); Kira Diemer and Xincheng Miao: equal (computations), writing (original draft); Ingo Fischer and Roland Mitric: supervision, writing (review and editing), project administration, funding aquisition; all other authors: investigation and data analysis (equal), writing (review and editing).

## Conflicts of interest

There are no conflicts to declare.

## Supplementary Material

SC-OLF-D5SC03745J-s001

SC-OLF-D5SC03745J-s002

## Data Availability

Additional experimental and computational data supporting this article have been included as part of the supplementary information (SI). Further data is available from the corresponding authors upon reasonable request. Supplementary information is available. See DOI: https://doi.org/10.1039/d5sc03745j.
