# Supplementary material for: Time-resolved X-ray spectroscopy of phenanthridine: elucidating the photodynamics of a nitrogen-containing polycyclic aromatic hydrocarbon
Source: Chem Sci. 2025 Oct 20;16(47):22711–22. doi: 10.1039/d5sc03745j (PMC12573282; doi:10.1039/d5sc03745j)
Supplement: SC-016-D5SC03745J-s001 [file SC-016-D5SC03745J-s001.pdf]

Supplementary Information (SI) for Chemical Science.
This journal is © The Royal Society of Chemistry 2025

Supporting Information for
Time-Resolved X-Ray Spectroscopy of Phenanthridine: Elucidating the Photodynamics of a Nitrogen-Containing Polycyclic Aromatic Hydrocarbon

Dorothee Schaffner,[a] Kira Diemer,[a] Xincheng Miao,[a] Emil Karaev,[a] Marco Flock,[a] Katharina Theil,[a] Constant Schouder,[b] Audrey Scognamiglio,[b] Lou Barreau,[b] Lionel Poisson,[b] Dennis Mayer,[c] Andre Al Haddad,[d] Antoine Sarracini,[d] Gregor Knopp,[d] Xinhua Xie,[d] Patrick Hemberger,[d] Kirsten Schnorr,[d] Roland Mitric,[*a] and Ingo Fischer[*a]

November 4, 2025

[a] Institute of Physical and Theoretical Chemistry, University of Würzburg, Am Hubland, 97074 Würzburg, Germany. E-mail: roland.mitric@uni-wuerzburg.de; ingo.fischer@uni-wuerzburg.de
[b]Université Paris-Saclay, CNRS, Institut des Sciences Moléculaires d'Orsay, 91405 Orsay, France.
[c]Deutsches Elektronen-Synchrotron DESY, Notkestrasse 85, 22607 Hamburg, Germany.
[d]Laboratory for Femtochemistry and Synchrotron Radiation, Paul Scherrer Institut (PSI), 5232 Villigen, Switzerland.

Contents

S1 Natural Orbitals and Natural Transition Orbitals S2
S2 Computed UV-Vis Absorption Spectrum S4
S3 REMPI Spectrum S5
S4 TR-PES S6
S5 Trajectory Surface Hopping S10
S6 Diabatization S11
S7 Normal Mode Analysis S13
S8 State-selective time-resolved X-ray absorption spectra S18
S9 Comparison between EOM-CCSD and RASPT2 X-ray absorption spectra S18
S10 Global fitting S20

S1 Natural Orbitals and Natural Transition Orbitals

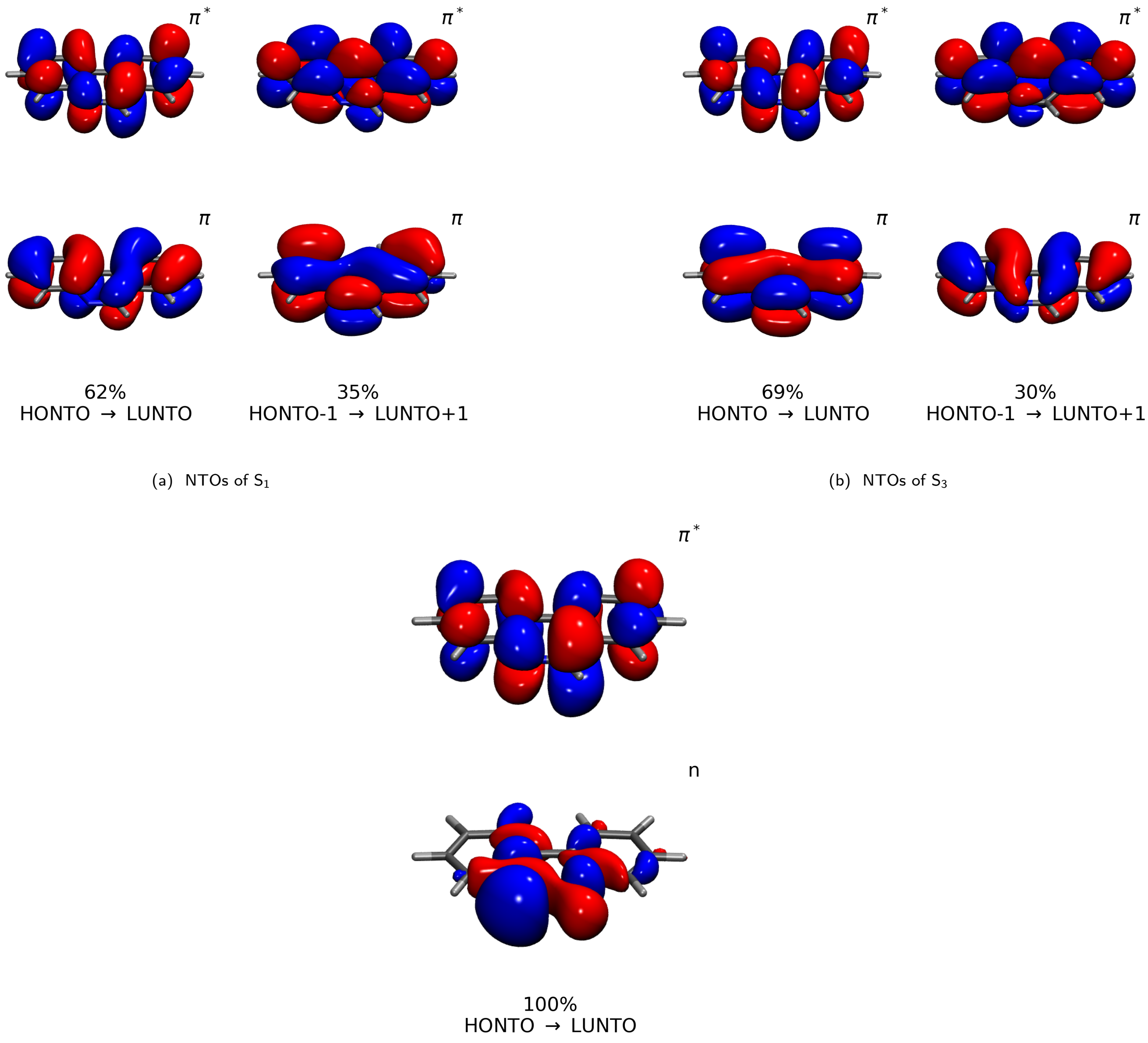

(a) NTOs of S_1

(b) NTOs of S_3

(c) NTOs of S_2

Fig. S1 Natural transition orbitals (NTOs) from the TD-ωB97X-D/def2-SV(P) calculation at the optimized ground state geometry of phenanthridine for the states S_1–S_3 along with the percentage participation in the electronic transition. The character is shown in the upper right corner. An isovalue of 0.02 was chosen.

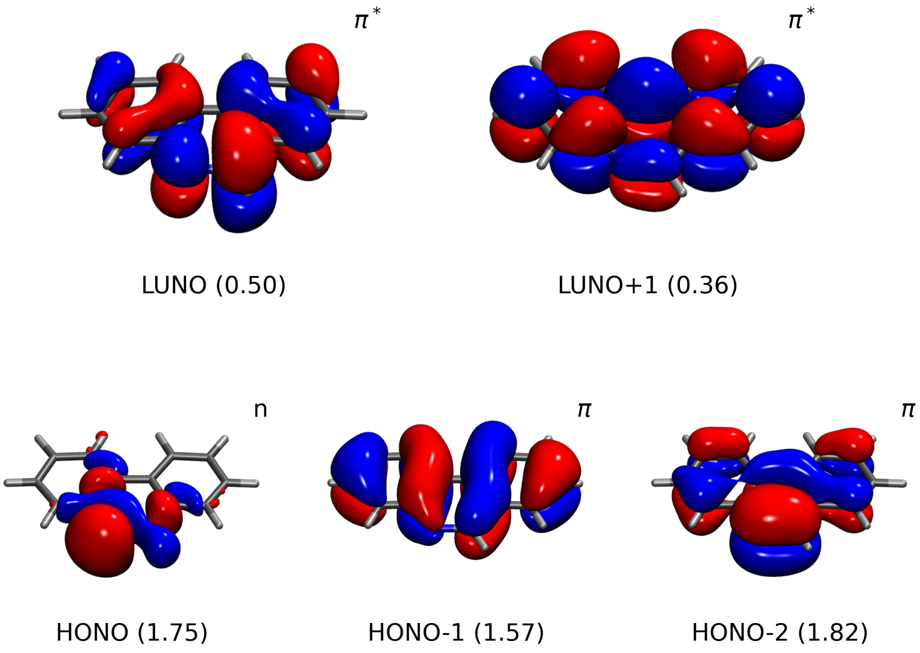

Fig. S2 Natural orbitals (NOs) from the SA4-RMS-CASPT2(6,5)/cc-pVDZ calculation at the optimized ground state geometry of phenanthridine. The occupation number is given in the parenthesis and the character is shown in the upper right corner. An isovalue of 0.02 was chosen.

S2 Computed UV-Vis Absorption Spectrum

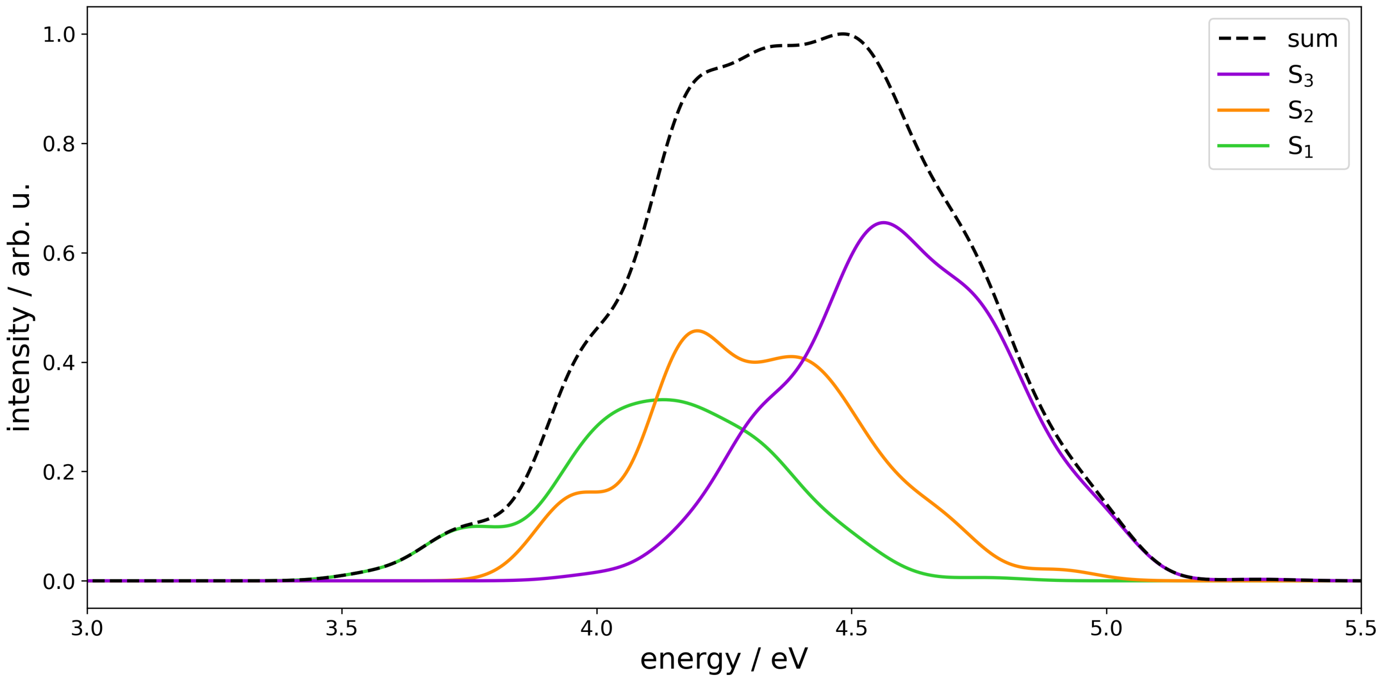

Fig. S3 Computed vertical UV-Vis absorption spectra of the three lowest excited singlet states S_1–S_3 averaged over 200 initial structures and broadened with a Gaussian profile of width $\sigma = 0.1$ eV. As can be seen, the transition into the S_3 state dominates the absorption spectrum at 268 nm (4.63 eV). The S_2 state, formally of $n\pi^*$ character, acquires intensity due to vibronic coupling.

S3 REMPI Spectrum

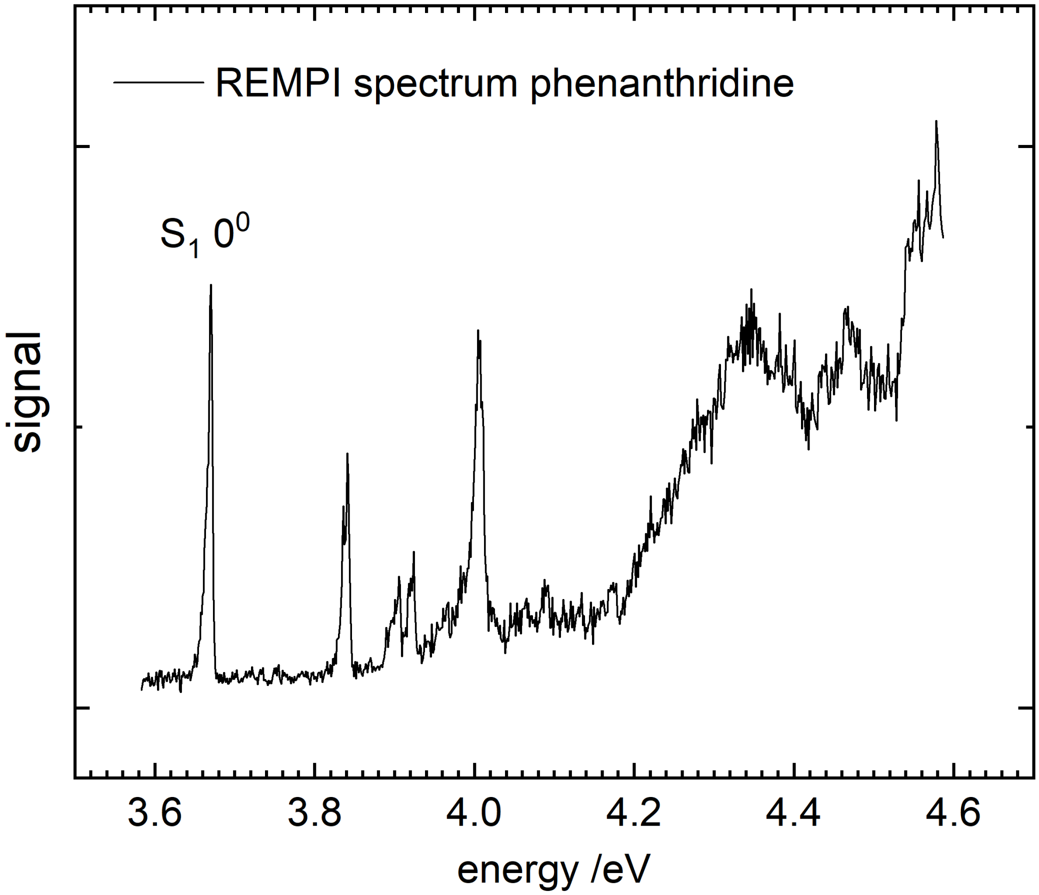

Fig. S4 Resonance-enhanced multiphoton ionization spectrum of phenanthridine, recorded with a ps-laser system. Band positions and assignments have been discussed in a previous publication.[1]

S4 TR-PES

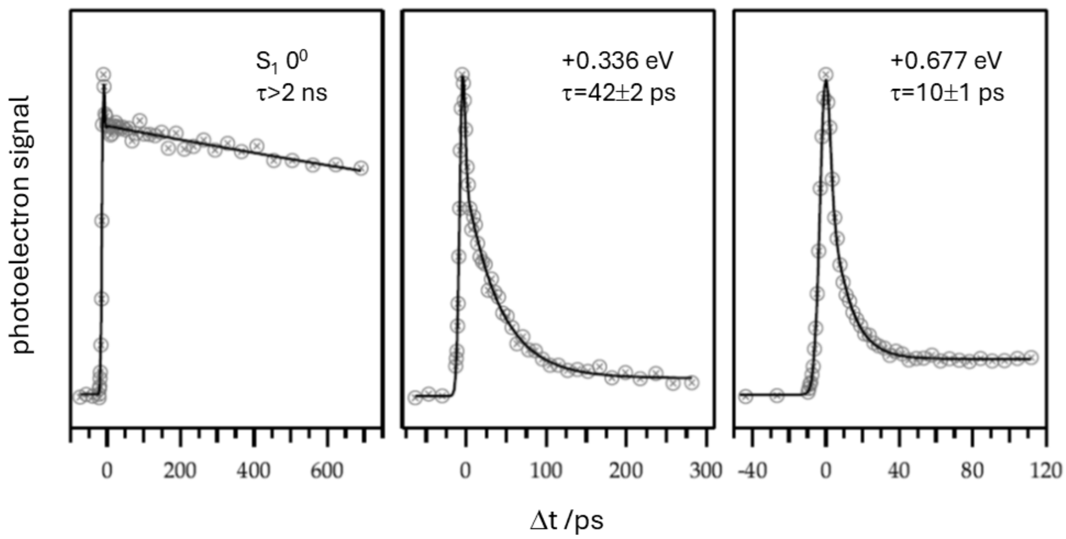

Fig. S5 Selected time delay traces of various zero-order states in the S_1 state of phenanthridine, obtained by time-resolved photoelectron spectroscopy.[2] The data have been recorded with a ps-laser system described previously.[3] As visible, decay traces are fitted by a monoexponential function with a single time constant τ.

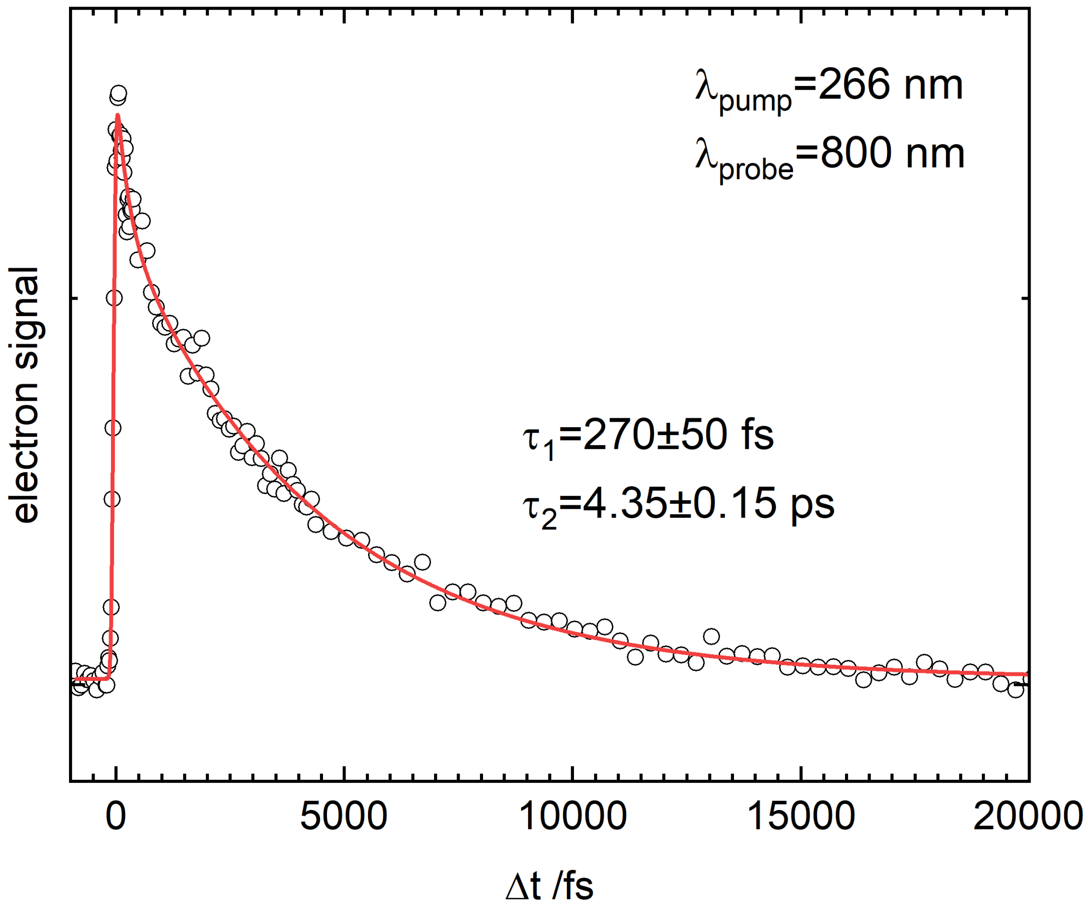

Fig. S6 Time delay trace of a laser photoelectron spectrum. The experimental setup has been described before.[4] Phenanthridine was excited at 266 nm, the dynamics was probed by multiphoton ionization at 800 nm. The fit (solid red line) yielded time constants of 270 fs and 4.35 ps.

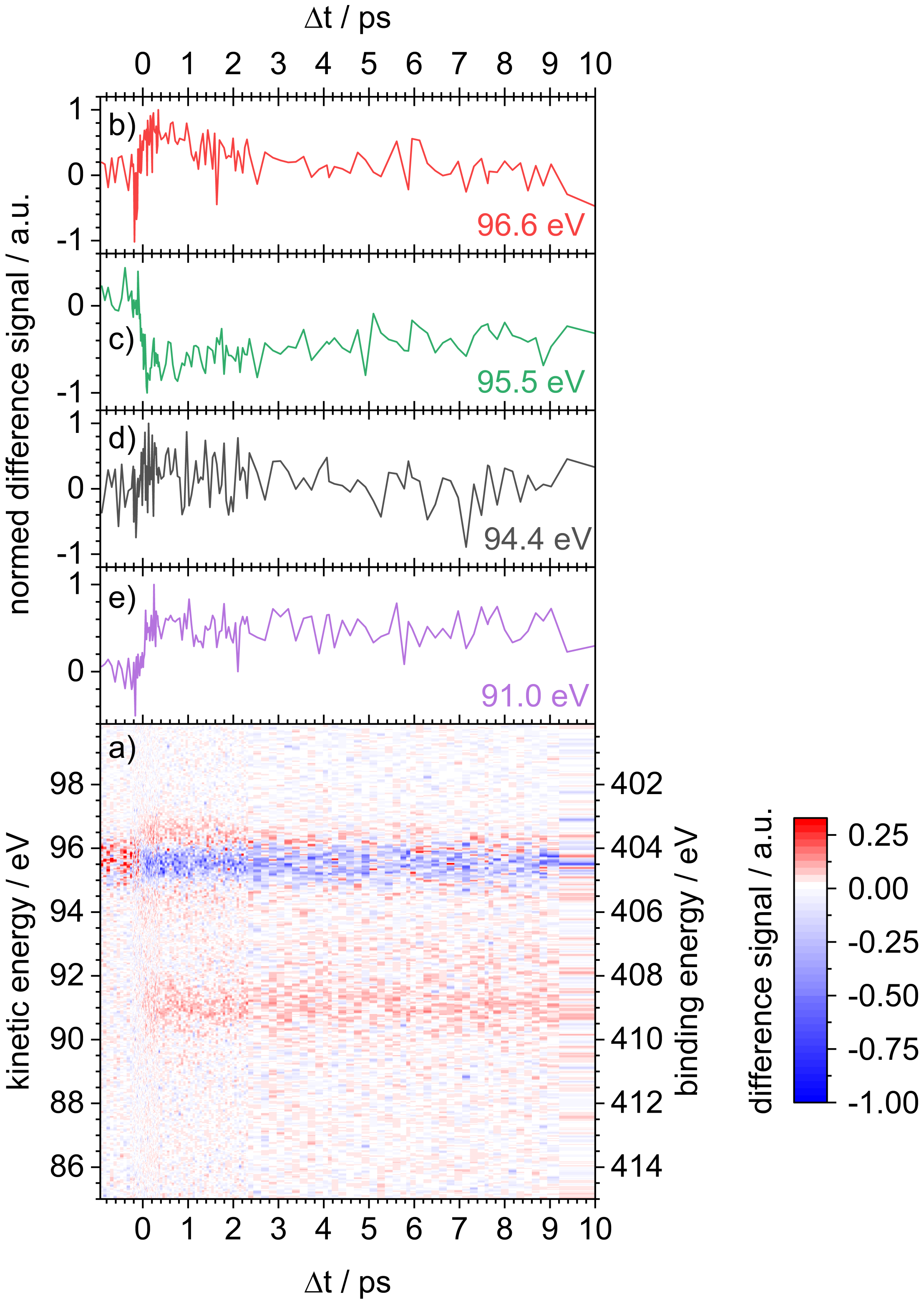

Fig. S7 (a) TR-XPS 2D map of phenanthridine recorded at $h\nu_{pump} = 4.63\,\mathrm{eV}$ $(268\,\mathrm{nm})$ and $h\nu_{probe} = 500\,\mathrm{eV}$, using a pump laser pulse energy of $20\,\mu\mathrm{J}$. Note that $h\nu_{probe}$ differs by $1\,\mathrm{eV}$ from the value employed in the TR-XPS 2D map shown in the main paper. (b)–(e) Delay traces for selected kinetic energies (eKE) in the 2D map.

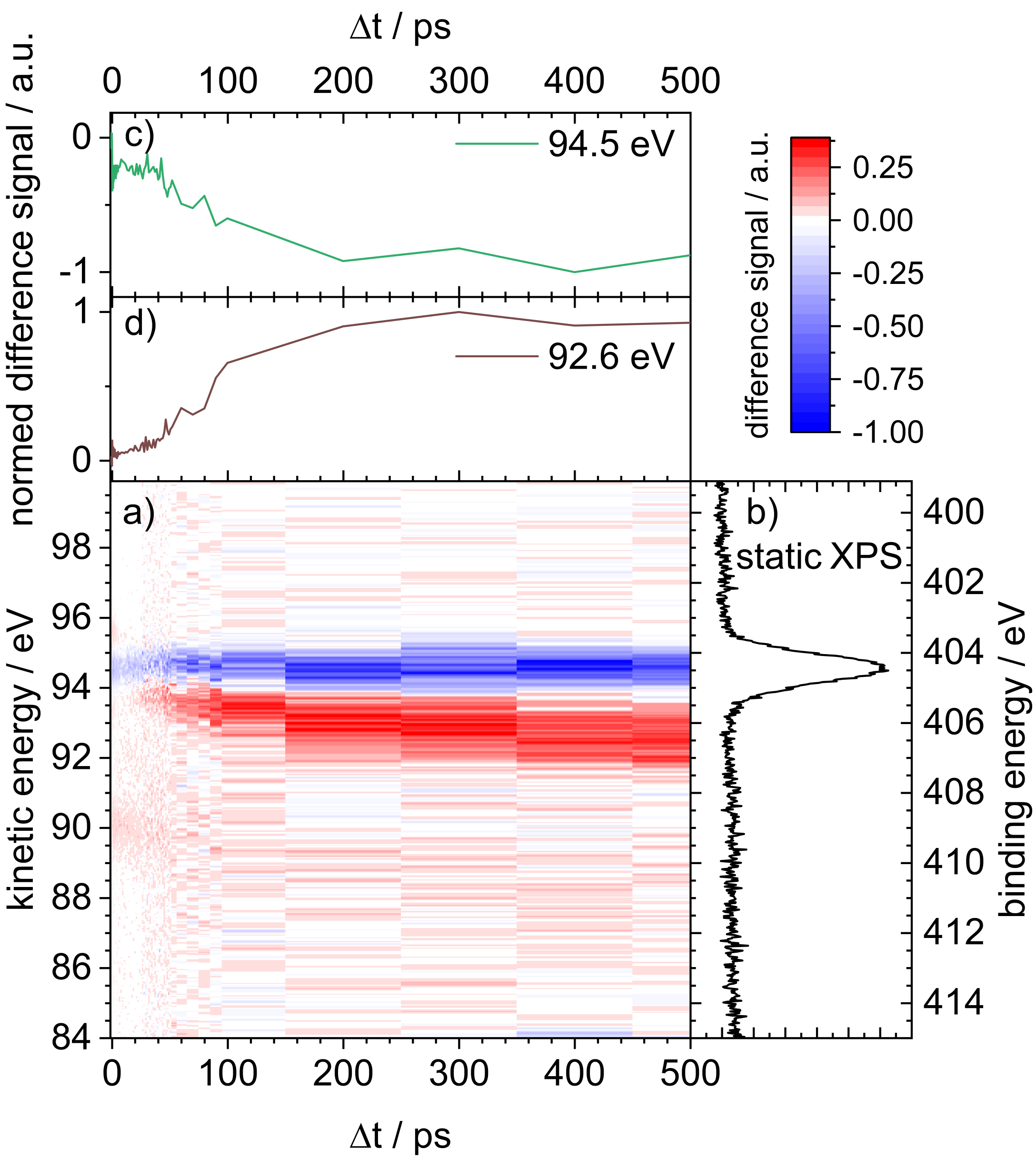

Fig. S8 (a) TR-XPS 2D map for delay times shown up to 500 ps. The spectrum was recorded at $h\nu_{pump} = 4.63\,\mathrm{eV}$ $(268\,\mathrm{nm})$ and $h\nu_{probe} = 499\,\mathrm{eV}$, using a pump laser pulse energy of $30\,\mu\mathrm{J}$. A band with an increasing red shift is visible that shows E_B = 406.4 eV at late delay times. It is assigned to the HCN photoproduct of a unimolecular fragmentation reaction. (b) Static XPS spectrum of phenanthridine. (c) Delay trace for the ground state bleach signal at around eKE = 94.5 eV. The ground state bleach increases again after 10 ps. (d) Delay trace for the HCN photoproduct at around eKE = 96.4 eV.

S5 Trajectory Surface Hopping

For trajectory-based nonadiabatic dynamics simulations with four electronic states, Tully's fewest-switches surface hopping[5] procedure in combination with the local diabatization scheme[6,7] using the TD-ωB97X-D/def2-SV(P) electronic structure method was performed.[8] Within this approach, the nuclei are treated classically by integrating the Newtonian equation of motion using the Velocity-Verlet algorithm[9], whereas the electron dynamics is addressed by solving the time-dependent Schrödinger equation

$$\mathrm{i}\hbar\dot{c}_i(t) = \sum_j H_{ij}(\vec{R}(t))c_j(t) - \mathrm{i}\hbar\sum_j D_{ij}(\vec{R}(t))c_j(t)\,. \tag{S.1}$$

In equation S.1, $c_i(t)$ represents the expansion coefficient of the electronic wavefunction for the ith electronic state and H_{ij} shortens the matrix element of the electronic Hamiltonian with respect to the electronic states i and j. The abbreviation D_{ij} stands for the scalar nonadiabatic coupling between the electronic states i and j, defined as

$$D_{ij}(\vec{R}(t)) = \underbrace{\langle\psi_i(\vec{R}(t))|\vec{\nabla}_R|\psi_j(\vec{R}(t))\rangle}_{\vec{d}_{ij}} \cdot \frac{\mathrm{d}\vec{R}(t)}{\mathrm{d}t}\,. \tag{S.2}$$

By solving equation S.1, the electronic state populations $\rho_{ii} = c_i^* c_i$ could be calculated, which in turn serves for predicting the probability of depopulating state i ($P_{i,\text{depopulation}}$) and the one for populating state j ($P_{j,\text{population}}$), calculated as follows:

$$P_{i,\text{depopulation}} = \Theta(-\dot{\rho}_{ii})\frac{-\dot{\rho}_{ii}}{\rho_{ii}}\Delta t \tag{S.3a}$$

$$P_{j,\text{population}} = \frac{\Theta(\dot{\rho}_{jj})\dot{\rho}_{jj}}{\sum_k \Theta(\dot{\rho}_{kk})\dot{\rho}_{kk}}\,, \tag{S.3b}$$

where Θ is the Heaviside step function.[10] Since DFT fails to describe the region near the S_1-S_0 conical intersection, the hop is enforced if their energy difference becomes smaller than 0.1 a. u.

S6 Diabatization

The adiabatic states are the solution of the electronic Hamiltonian obtained in the electronic structure calculations. A diabatic basis with state characters having less dependence of $\vec{R}$ than those in the adiabatic basis can be chosen to analyze the time-dependent electronic character of the excited adiabatic states throughout the dynamics simulations by expanding them into the diabatic basis.[11,12] In this work, the trajectory surface hopping procedure was combined with the linear-response TDDFT electronic structure method. The different excited states I, J, etc. for $t > 0$ are thus described as a linear combination of singly excited configuration state functions (CSFs)

$$|\Psi_I\rangle = \sum_{ia} T^I_{ia} |\Phi_{ia}\rangle \ , \tag{S.4}$$

where the CSFs are given in the basis of the occupied and virtual molecular Kohn-Sham orbitals φ, indexed by i, j, etc. and a, b, etc., that are obtained in a ground state calculation at the respective geometry. The expansion coefficients T^I_{ia} form the transition density matrix $\boldsymbol{T^I}$ in the adiabatic basis. The diabatic basis is chosen as the CSFs of the optimized reference nuclear geometry $|\Phi_{\tilde{i}\tilde{a}}\rangle$ featuring the transformed orbitals $\tilde{\phi}$ so that the excited states in diabatic representation are given by

$$|\Psi_I\rangle = \sum_{\tilde{i}\tilde{a}} \tilde{T}^I_{\tilde{i}\tilde{a}} |\Phi_{\tilde{i}\tilde{a}}\rangle \tag{S.5}$$

with the diabatic transition density matrix $\boldsymbol{\tilde{T}^I}$. To reveal the adiabatic state composition with respect to the diabatic states at the reference geometry, i.e., $\boldsymbol{\tilde{T}^I}$, the occupied adiabatic MOs φ^{occ} are expanded in the occupied diabatic MOs $\tilde{\varphi}^{\mathrm{occ}}$ according to

$$\varphi^{\mathrm{occ}}_i = \sum_{\tilde{j}} C^{\mathrm{occ}}_{\tilde{j}i} \tilde{\varphi}^{\mathrm{occ}}_{\tilde{j}} \ , \tag{S.6}$$

where $C^{\mathrm{occ}}_{\tilde{j}i}$ are the elements of the transformation matrix $\boldsymbol{C^{\mathrm{occ}}}$. Using the expression of the MOs in terms of the atomic orbitals η in the chosen basis set with the MO coefficient matrices $\boldsymbol{\tilde{c}^{\mathrm{occ}}}$ and $\boldsymbol{c^{\mathrm{occ}}}$,

$$\tilde{\varphi}^{\mathrm{occ}}_{\tilde{i}} = \sum_{\tilde{j}} c^{\mathrm{occ}}_{\tilde{j}\tilde{i}} \eta_{\tilde{j}} \text{ and } \varphi^{\mathrm{occ}}_i = \sum_j c^{\mathrm{occ}}_{ji} \eta_j \ , \tag{S.7}$$

allows for the evaluation of the transformation matrix $\boldsymbol{C^{\mathrm{occ}}}$ as

$$\langle \tilde{\varphi}^{\mathrm{occ}}_{\tilde{i}} | \varphi^{\mathrm{occ}}_j \rangle = \sum_{\tilde{k}l} \langle c^{\mathrm{occ}}_{\tilde{k}\tilde{i}} \eta_{\tilde{k}} | c^{\mathrm{occ}}_{lj} \eta_l \rangle = \sum_{\tilde{k}l} c^{\mathrm{occ}*}_{\tilde{k}\tilde{i}} \langle \eta_{\tilde{k}} | \eta_l \rangle \, c^{\mathrm{occ}}_{lj} \tag{S.8a}$$

$$= ((\boldsymbol{\tilde{c}^{\mathrm{occ}}})^\dagger \boldsymbol{S} \boldsymbol{c^{\mathrm{occ}}})_{\tilde{i}j} = C^{\mathrm{occ}}_{\tilde{i}j} \tag{S.8b}$$

with the overlap matrix elements $S_{\tilde{k}l} = \langle \eta_{\tilde{k}} | \eta_l \rangle$. The matrix $\boldsymbol{C^{\mathrm{virt}}}$ is constructed analogously; both are subsequently unitarized by employing the Löwdin orthogonalization[13] procedure. Finally, the ansatz

$$\tilde{T}^I_{\tilde{i}\tilde{a}} = \sum_{jb} C^{\mathrm{occ}}_{\tilde{i}j} T^I_{jb} C^{\mathrm{virt}}_{\tilde{a}b}{}^* = \sum_{jb} C^{\mathrm{occ}}_{\tilde{i}j} T^I_{jb} C^{\mathrm{virt}}_{b\tilde{a}} = (\boldsymbol{C^{\mathrm{occ}}} \boldsymbol{T^I} (\boldsymbol{C^{\mathrm{virt}}})^\dagger)_{\tilde{i}\tilde{a}} \tag{S.9}$$

yields the transformed transition density matrix $\boldsymbol{\tilde{T}^I}$ in the diabatic basis.[14,15]

Here, the molecular orbitals of the optimized electronic ground state geometry were used to construct the diabatic basis. To reduce

the computational effort, the transition density matrix $\boldsymbol{T}^{\boldsymbol{I}}$ for all trajectories was determined every femtosecond within the first 20 fs, then every 5 fs up to 100 fs and then every 10 fs. The contribution for a certain excitation type was examined by summing up the squared elements from the CI coefficient matrix $\tilde{\boldsymbol{T}}^{\boldsymbol{I}}$ for the primarily contributing orbitals.

S7 Normal Mode Analysis

A polyatomic molecule with N_{nuc} nuclei is described by $3N_{\mathrm{nuc}}$ cartesian coordinates stored in the vector $\vec{x}$ with the elements x_i with $i = 1, 2, \ldots, 3N_{\mathrm{nuc}}$. Each mode of motion is to be considered so that any displacements $\vec{x}_{\mathrm{rel}} = \vec{x} - \vec{x}_0$ with respect to the equilibrium structure defined by $\vec{x}_0$ are treated. First, mass-weighted coordinates q_i are introduced as

$$\vec{q} = \boldsymbol{M}^{\frac{1}{2}} \vec{x}_{\mathrm{rel}} \, , \tag{S.10}$$

where $\boldsymbol{M}$ represents the $3N_{\mathrm{nuc}}$-dimensional diagonal matrix containing the masses of the nuclei. First derivative in time leads to the corresponding velocity vector $\dot{\vec{q}} = \boldsymbol{M}^{\frac{1}{2}} \dot{\vec{x}}_{\mathrm{rel}}$ with $\dot{\vec{x}}_{\mathrm{rel}} = \dot{\vec{x}} - \dot{\vec{x}}_0$.[16] The total energy E of a polyatomic molecule is given by the sum of kinetic energy T and potential energy V. The former can be written as

$$T = \frac{1}{2} \sum_{i=1}^{3N_{\mathrm{nuc}}} M_i (\dot{x}_i^{\mathrm{rel}})^2 = \frac{1}{2} \sum_{i}^{3N_{\mathrm{nuc}}} \dot{x}_i^{\mathrm{rel}} M_i^{\frac{1}{2}} M_i^{\frac{1}{2}} \dot{x}_i^{\mathrm{rel}} = \frac{1}{2} \dot{\vec{x}}_{\mathrm{rel}}^{T} \boldsymbol{M}^{\frac{1}{2}} \boldsymbol{M}^{\frac{1}{2}} \dot{\vec{x}}_{\mathrm{rel}} = \frac{1}{2} \dot{\vec{q}}^{T} \dot{\vec{q}}, \tag{S.11}$$

whereas the latter can be expanded into a Taylor series up to the second order around the reference structure at the potential energy minimum, i.e.,

$$V \approx V(\vec{x}_0) + \sum_{i}^{3N_{\mathrm{nuc}}} \left. \frac{\partial V}{\partial x_i} \right|_{x_i = x_{i,0}} + \frac{1}{2} \sum_{ij}^{3N_{\mathrm{nuc}}} \left. \frac{\partial^2 V}{\partial x_i \partial x_j} \right|_{x_i = x_{i,0},\, x_j = x_{j,0}} x_i^{\mathrm{rel}} x_j^{\mathrm{rel}} \, . \tag{S.12}$$

In this representation, the potential energy depends on all the displacements of the atoms from their equilibrium positions. While the first term is constant and can be set equal to zero, the first derivatives of the potential energy are all zero at the equilibrium, so that equation S.12 can be simplified to

$$V \approx \frac{1}{2} \sum_{ij}^{3N_{\mathrm{nuc}}} K_{ij} x_i^{\mathrm{rel}} x_j^{\mathrm{rel}} = \frac{1}{2} \vec{x}_{\mathrm{rel}}^{T} \boldsymbol{K} \vec{x}_{\mathrm{rel}} = \frac{1}{2} \vec{q}^{T} \boldsymbol{M}^{-\frac{1}{2}} \boldsymbol{K} \boldsymbol{M}^{-\frac{1}{2}} \vec{q} \tag{S.13a}$$

$$K_{ij} = \left. \frac{\partial^2 V}{\partial x_i \partial x_j} \right|_{x_i = x_{i,0},\, x_j = x_{j,0}} \tag{S.13b}$$

using the mass-weighted coordinates and the generalized force constants K_{ij}, which form the Hessian matrix $\boldsymbol{K}$.[16] From this, the mass-weighted Hessian $\boldsymbol{K_M}$ is defined and diagonalized according to

$$\boldsymbol{K_M} = \boldsymbol{M}^{-\frac{1}{2}} \boldsymbol{K} \boldsymbol{M}^{-\frac{1}{2}} \tag{S.14a}$$

$$\boldsymbol{K_M} = \boldsymbol{L} \boldsymbol{D} \boldsymbol{L}^{T} \, . \tag{S.14b}$$

The matrix $\boldsymbol{L}$ contains the orthogonal and normalized eigenvectors as columns, which are the coordinates of the normal modes. The diagonal matrix $\boldsymbol{D}$ has the eigenvalues λ_i as elements. Substituting this in equation S.13a yields

$$V \approx \frac{1}{2} \vec{q}^{T} \boldsymbol{K_M} \vec{q} = \frac{1}{2} \vec{q}^{T} \boldsymbol{L} \boldsymbol{D} \boldsymbol{L}^{T} \vec{q} = \frac{1}{2} \vec{Q}^{T} \boldsymbol{D} \vec{Q} = \frac{1}{2} \sum_{i}^{3N_{\mathrm{nuc}}} \lambda_i Q_i^2 \, , \tag{S.15}$$

where the normal coordinates

$$\vec{Q} = \boldsymbol{L}^{T} \vec{q} = \boldsymbol{L}^{T} \boldsymbol{M}^{\frac{1}{2}} \vec{x}_{\mathrm{rel}} \tag{S.16}$$

have been introduced. Equation S.16 defines the transformation of cartesian coordinates to normal coordinates. Expressing the kinetic energy contribution (equation S.11) in terms of normal coordinates by writing

$$T = \frac{1}{2}\dot{\vec{q}}^T\dot{\vec{q}} = \frac{1}{2}\dot{\vec{q}}^T\boldsymbol{L}\boldsymbol{L}^T\dot{\vec{q}} = \frac{1}{2}\dot{\vec{Q}}^T\dot{\vec{Q}} = \frac{1}{2}\sum_i^{3N_{\text{nuc}}}\dot{Q}_i^2\,, \tag{S.17}$$

the total energy of the polyatomic molecule reads

$$E = T + V = \frac{1}{2}\sum_i^{3N_{\text{nuc}}}(P_i^2 + \omega_i^2 Q_i^2) \tag{S.18}$$

after setting the momentum $P_i = \dot{Q}_i$ and the eigenfrequencies $\omega_i^2 = \lambda_i$. Thus, the total energy can be expressed as a sum of independent harmonic oscillators of unit mass and frequency ω_i. Of the total $3N_{\text{nuc}}$ degrees of freedom, six modes will have an eigenvalue λ_i close to zero in a non-linear molecule because displacement along these normal coordinates corresponds to translations and rotations.[16]

Figure S9 reveals that several modes are excited during the dynamics simulations, of which the modes labeled by ν_1, ν_6, ν_{17}, and ν_{24} show the largest displacement on average. The first is assigned to an out-of-plane bending movement, while the other three correspond to in-plane bendings. The vibrational modes are depicted in Figure S10. To obtain cuts of the potential energy surfaces of the three lowest excited singlet states along selected vibrational normal modes, the displacement along a normal mode $\vec{L}_i$ of the equilibrium structure (columns of $\boldsymbol{L}$ in equation S.14b) was simulated according to

$$\vec{x} = \vec{x}_0 + \frac{\boldsymbol{L}}{\sqrt{\boldsymbol{M}}}\vec{Q}\,, \tag{S.19}$$

where just the ith element of $\vec{Q}$ is non-zero so that the mass-weighted displacements are evaluated to

$$\Delta x \cdot \sqrt{M_i} = (\vec{x} - \vec{x}_0) \cdot \sqrt{M_i} = \vec{L}_i Q_i\,. \tag{S.20}$$

The results are depicted in Figure S11. Notice that no crossings of the PESs were observed in the scan along ν_1, so this low-frequency mode was expected to not play any roles in the relaxation process.

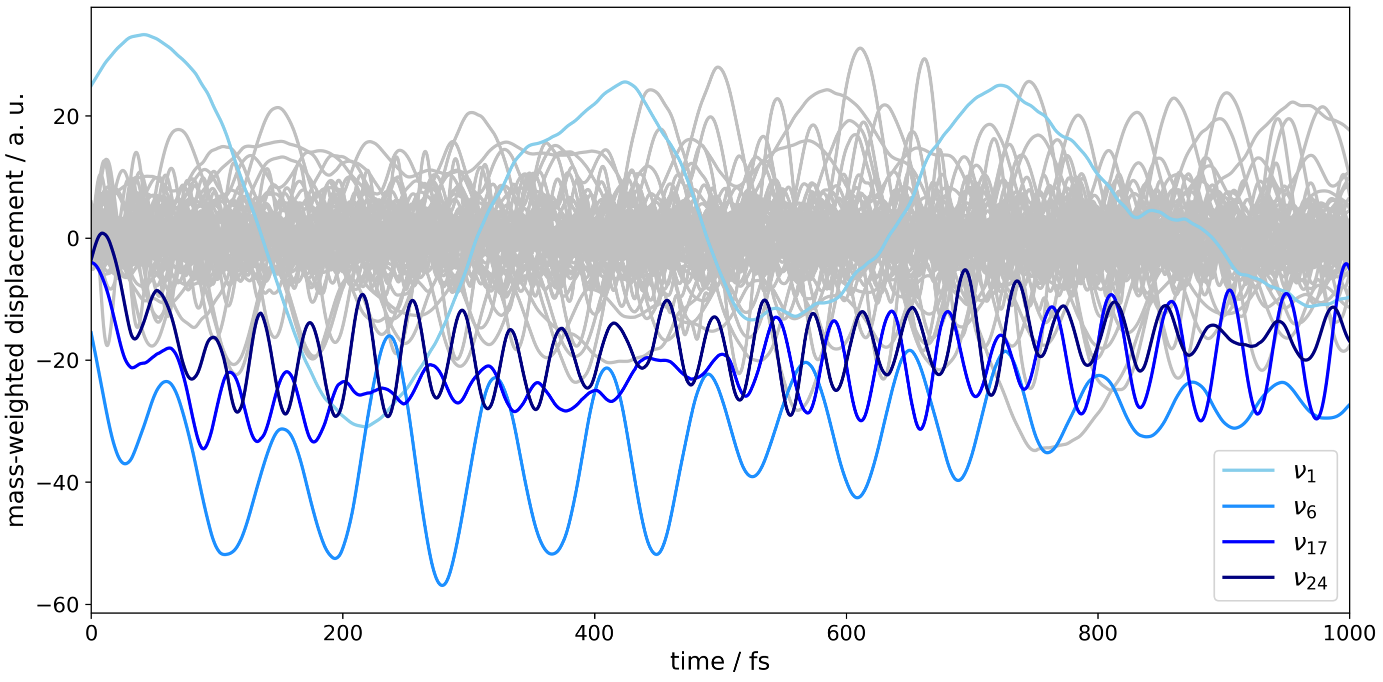

Fig. S9 Time-dependent normal mode displacements obtained by projection onto the equilibrium ground state normal coordinates of phenanthridine averaged over the 24 trajectories initially excited to S_3.

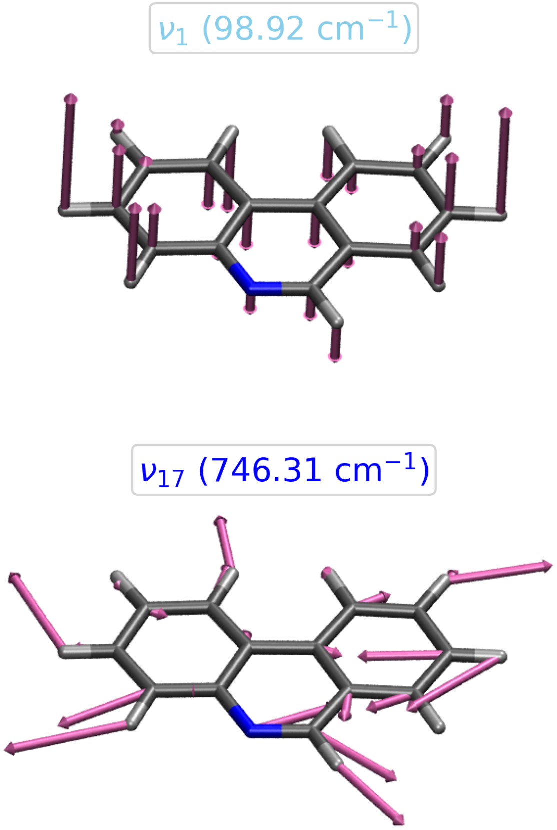

Fig. S10 Vibrational modes that are excited during the molecular dynamics simulations, namely the out-of-plane bending ν_1 and the in-plane bendings ν_6, ν_{17}, and ν_{24}.

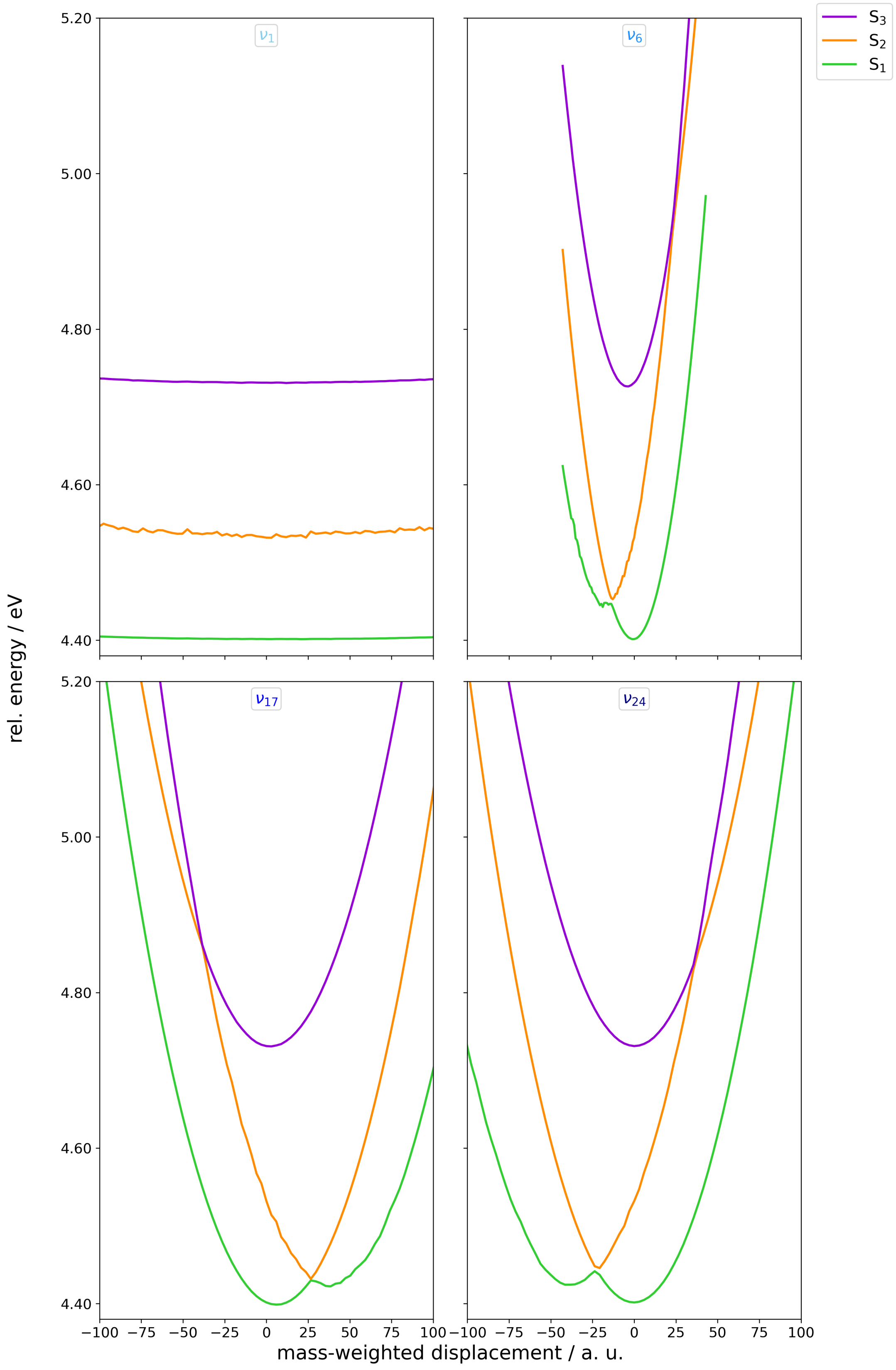

Fig. S11 Cuts of the potential energy surfaces along selected vibrational normal modes for the three lowest excited singlet states of phenanthridine.

S8 State-selective time-resolved X-ray absorption spectra

Figure S12 shows the contributions of the transient population in the S_0, S_1, S_2 and S_3 state of the simulated time-resolved XAS at RASPT2 level of theory. Each spectrogram was normalized individually. The ground state XAS was subtracted from each trace to provide a reference to the location of the GSB signal. The ensemble ground state spectrum was broadened with a Gaussian profile with the width of $\sigma_{\mathrm{exc}} = 0.1\,\mathrm{eV}$, while the transient TR-XAS was broadened with a two-dimensional Gaussian profile with the standard deviations of $\sigma_{\mathrm{exc}} = 0.1\,\mathrm{eV}$ and $\sigma_{\mathrm{t}} = 20\,\mathrm{fs}$. The rapid decay in S_3 and S_2 is easily recognized, as well as the buildup of hot ground state (see upper left trace of S_0).

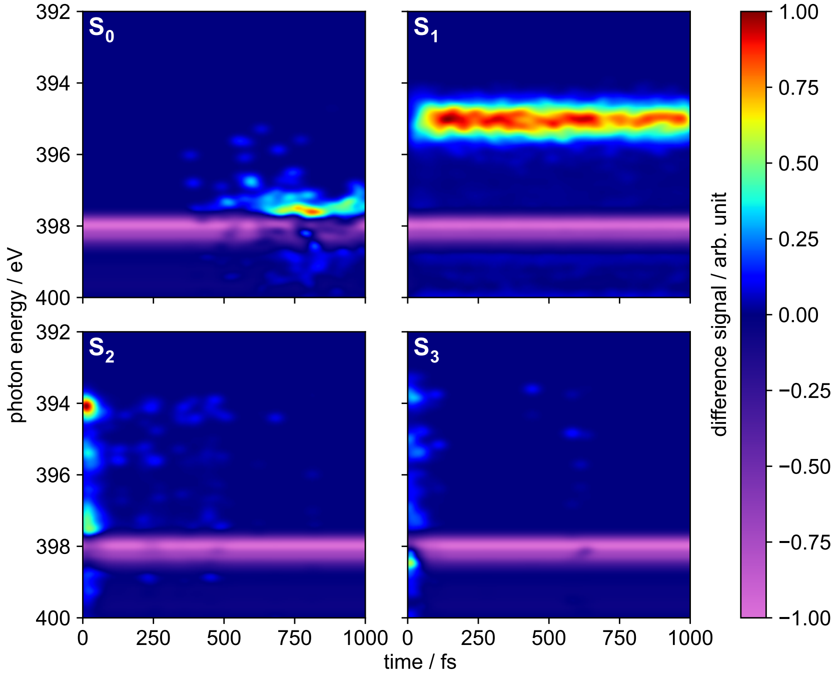

Fig. S12 Simulated transient X-ray absorption spectra for the S_3, S_2, S_1 and S_0 states of phenanthridine computed using the RASPT2 method.

S9 Comparison between EOM-CCSD and RASPT2 X-ray absorption spectra

This section aims at comparing the simulated X-ray absorption spectra calculated at EOM-CCSD and RASPT2 level of theory. We therefore compare in more detail (see Figure 6a) in the main paper) the simulated X-ray absorption spectra at the ground state equilibrium structure of **1** computed at EOM-CCSD (dashed line, Figure S13b) and RASPT2 (solid line, Figure S13c) level of theory with the experimental spectrum (dotted line, Figure S13a). As described in the main paper the EOM-CCSD simulation was shifted by $-4.0\,\mathrm{eV}$ so that the 1s $\rightarrow$ LUMO absorption band (A) coincides with the most intense band in the experimental spectrum. The computed spectrum is in good agreement with the experimental one for the signal at ca. $401.3\,\mathrm{eV}$ (B), which can be assigned to the mixed transition involving 1s $\rightarrow$ LUMO+1 (55%) and 1s $\rightarrow$ LUMO+2 (22%) orbital excitations. The RASPT2 simulation was shifted by $-3.1\,\mathrm{eV}$ analogously. Due to the absence of the LUMO+2 in its active space, the band B is not well reproduced in the RASPT2 spectrum and appears at $399.6\,\mathrm{eV}$ (B′). In order to compare EOM-CCSD and RASPT2 results also for the excited states, the right hand side of Figure S13 displays the simulated XAS of the valence excited states S_1 to S_3 at the ground state equilibrium structure of **1**. Only the transitions on the low energy side of the GSB will be analysed in the following. The S_1 state (Figure S13d), which has $1\pi\pi^*$ character at the S_0 equilibrium geometry, gives rise to absorption bands caused by the 1s $\rightarrow \pi$ hole-filling transition, which are located at $394.0\,\mathrm{eV}$ and $397.2\,\mathrm{eV}$ (C) in

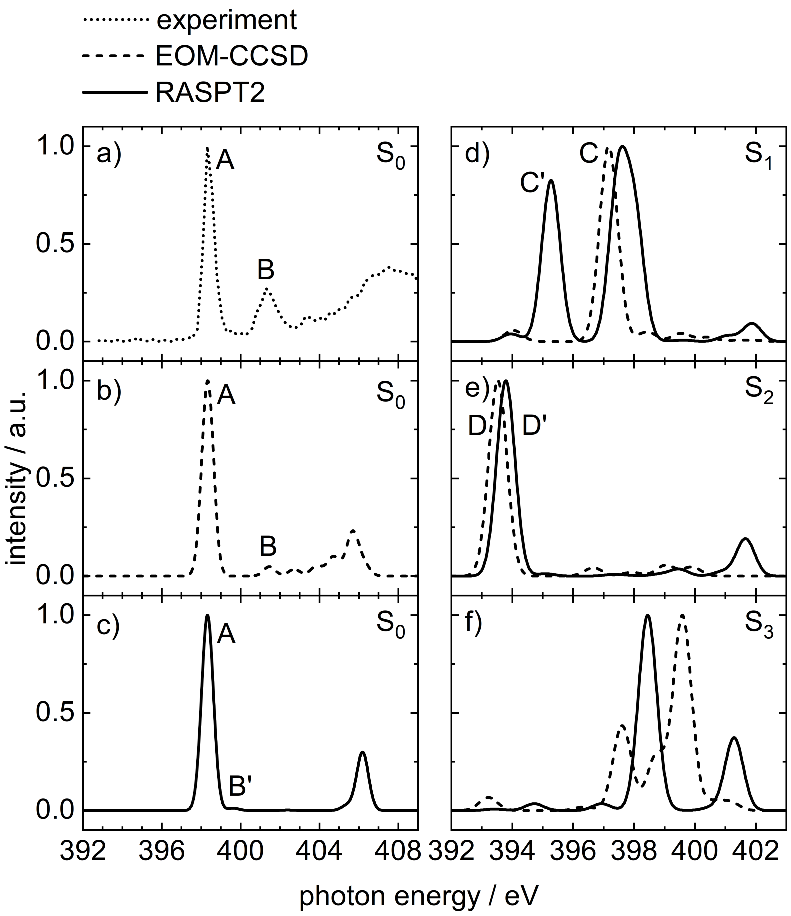

Fig. S13 Comparison of the experimental static XAS (a) with the simulated XAS for the electronic ground state computed using EOM-CCSD (b) and RASPT2 (c). (d)–(f) Simulated XAS for the three lowest excited singlet states. All calculations were carried out at the equilibrium structure of 1 and the simulated stick spectra were broadened with a Gaussian profile with the width of $\sigma = 0.3\,\mathrm{eV}$. The EOM-CCSD (dashed) and RASPT2 (solid) spectra were shifted by $-4.0\,\mathrm{eV}$ and $-3.1\,\mathrm{eV}$, respectively.

the EOM-CCSD spectrum. The first of these two signals was assigned to a target state, in which the LUMO is singly occupied, whereas the final configuration of the second signal has mixed contributions of both LUMO+1 and LUMO+2 orbitals. These signals are found at $393.9\,\mathrm{eV}$ and $395.3\,\mathrm{eV}$ (C′) in the RASPT2 calculation, i.e., a shift of $1.9\,\mathrm{eV}$ was observed for absorption band C/C′ between the two methods. This is explained by the missing of the LUMO+2 in the active space of the RASPT2 approach. The signal at $397.5\,\mathrm{eV}$ in the RASPT2 calculation corresponds to 1s $\rightarrow$ π* excitations, that cannot be described adequately by EOM-CCSD, because the final states correspond to double excitations with respect to the ground state. X-ray excitation of the nπ* state (Figure S13e) produces an absorption band due to 1s $\rightarrow$ n transition, which appears at $393.5\,\mathrm{eV}$ (D) in the EOM-CCSD simulation and at $393.8\,\mathrm{eV}$ (D′) in the RASPT2 simulation. The static XAS of the second $\pi\pi$* state (Figure S13e) features absorption bands energetically close to those of the first $\pi\pi$* state and ground state. Note that the spectra are separately normalized. The oscillator strengths of the most intense peaks in the EOM-CCSD computations are 0.0013, 0.0067, 0.0003 for the excitation from S_1, S_2, S_3, respectively. For the RASPT2 simulation, the corresponding values are 0.0112, 0.0302, 0.0290. Albeit the energetic shift of $1.9\,\mathrm{eV}$ of transitions related to LUMO+2, RASPT2 offers a qualitatively correct description of all relevant transitions. Furthermore, it can describe multiply excited states resulted from the X-ray excitation, in contrary to EOM-CCSD. Therefore, RASPT2 was used to simulate the TR-XAS as given in the main paper.

S10 Global fitting

The global fitting was performed following the procedure described in Ref. 17 but applied here either to the TR-XPS signal or to the TR-XAS signal. Basically, a set of functions, solutions of a sequential relaxation mechanism, is built taking into account a gaussian temporal profile for the instrument response function and assuming three parameters: the time zero t_0 and two time decays τ_1 and τ_2 [4]. The experimental difference signal is algebraically projected simultaneously onto this set of functions to adjust it onto this basis-set and provide a residue. Then a standard Levenberg–Marquardt procedure is applied to this procedure to minimize the residue by optimizing t_0, τ_1 and τ_2. It is also fitting the whole set of data together.

References

1 F. Sturm, C. Herok and I. Fischer, *J. Phys. Chem. A*, 2024, **128**, 8421–8427.

2 M. Flock, *Doctoral thesis*, Universität Würzburg, 2021.

3 M. Flock, L. Bosse, D. Kaiser, B. Engels and I. Fischer, *Phys. Chem. Chem. Phys.*, 2019, **21**, 13157–13164.

4 J.-M. Mestdagh, L. Barreau and L. Poisson, *Phys. Chem. Chem. Phys.*, 2024, **26**, 11516–11530.

5 J. C. Tully, *J. Chem. Phys.*, 1990, **93**, 1061–1071.

6 G. Granucci, M. Persico and A. Toniolo, *J. Chem. Phys.*, 2001, **114**, 10608–10615.

7 F. Plasser, G. Granucci, J. Pittner, M. Barbatti, M. Persico and H. Lischka, *J. Chem. Phys.*, 2012, **137**, 22A514.

8 R. Mitrić, U. Werner and V. Bonačić-Koutecký, *J. Chem. Phys.*, 2008, **129**, 164118.

9 W. C. Swope, H. C. Andersen, P. H. Berens and K. R. Wilson, *J. Chem. Phys.*, 1982, **76**, 637–649.

10 P. G. Lisinetskaya and R. Mitrić, *Phys. Rev. A*, 2011, **83**, 033408.

11 V. Bonačić-Koutecký and R. Mitrić, *Chem. Rev.*, 2005, **105**, 11–66.

12 A. Mandal, S. S. Yamijala and P. Huo, *J. Chem. Theory Comput.*, 2018, **14**, 1828–1840.

13 P.-O. Löwdin, *Advances in Quantum Chemistry*, Academic Press, 1970, vol. 5, pp. 185–199.

14 W. Liu, B. Lunkenheimer, V. Settels, B. Engels, R. F. Fink and A. Köhn, *J. Chem. Phys.*, 2015, **143**, 084106.

15 J. Hoche, M. Flock, X. Miao, L. N. Philipp, M. Wenzel, I. Fischer and R. Mitric, *Chem. Sci.*, 2021, **12**, 11965–11975.

16 P. Atkins and R. Friedman, *Molecular Quantum Mechanics*, Oxford University Press, 4th edn, 2008.

17 J.-M. Mestdagh and L. Poisson, *ChemPhysChem*, 2020, **21**, 2605–2613.
