# Supplementary material for: Time-resolved X-ray spectroscopy of phenanthridine: elucidating the photodynamics of a nitrogen-containing polycyclic aromatic hydrocarbon
Source: Chem Sci. 2025 Oct 20. Online ahead of print. doi: 10.1039/d5sc03745j (PMC12573282; doi:10.1039/d5sc03745j)

Supplementary Information (SI) for Chemical Science.
This journal is © The Royal Society of Chemistry 2025

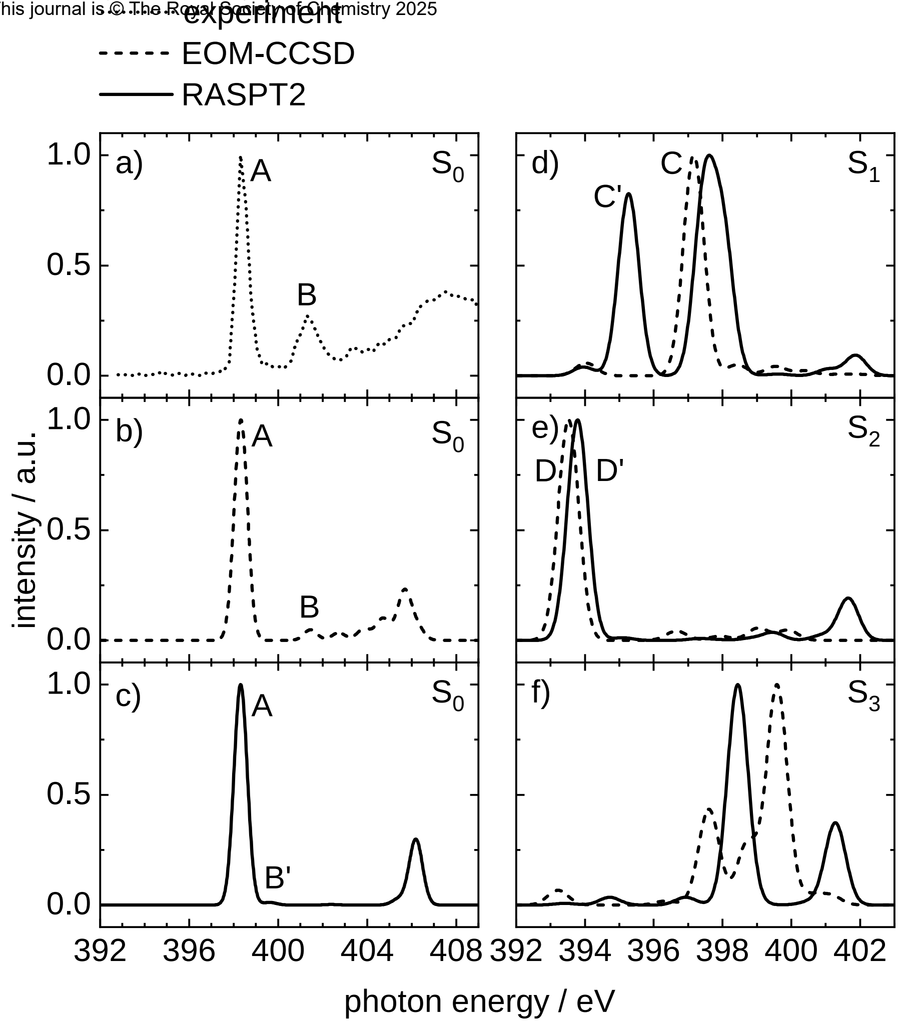

Supplement: Supplementary file 1 [file SC-OLF-D5SC03745J-s001.pdf]
